# Physical Activity and Health-Related Quality of Life in Adults With a Neurologically-Related Mobility Disability During the COVID-19 Pandemic: An Exploratory Analysis

**DOI:** 10.3389/fneur.2021.699884

**Published:** 2021-08-27

**Authors:** Tom E. Nightingale, Nicola R. Heneghan, Sally A. M. Fenton, Jet J. C. S. Veldhuijzen van Zanten, Catherine R. Jutzeler

**Affiliations:** ^1^School of Sport, Exercise and Rehabilitation Sciences, University of Birmingham, Birmingham, United Kingdom; ^2^International Collaboration on Repair Discoveries (ICORD), University of British Columbia, Vancouver, BC, Canada; ^3^Centre of Precision Rehabilitation for Spinal Pain (CPR Spine), School of Sport, Exercise and Rehabilitation Sciences, University of Birmingham, Birmingham, United Kingdom; ^4^Medical Research Council-Versus Arthritis Centre for Musculoskeletal Ageing, University of Birmingham, Birmingham, United Kingdom; ^5^Department of Biosystems Science and Engineering, Swiss Federal Institute of Technology (ETH) Zurich, Zurich, Switzerland; ^6^SIB Swiss Institute of Bioinformatics, Ecublens, Switzerland

**Keywords:** SARS-CoV-2, exercise, neurological disorders, well-being, pandemic, mental health

## Abstract

**Background:** During the coronavirus-19 (COVID-19) pandemic various containment strategies were employed. Their impact on individuals with neurological conditions, considered vulnerable to COVID-19 complications, remains to be determined.

**Objective:** To investigate associations between physical activity and health-related quality of life outcomes in individuals with a neurological condition during government mandated COVID-19 restrictions.

**Methods:** An e-survey assessing fear of COVID-19, physical activity level and health-related quality of life outcomes (functional disability and pain, anxiety and depression, loneliness, fatigue, and vitality) was distributed to individuals with a neurologically-related mobility disability living in the United Kingdom. Open-ended questions were also included to contextualize barriers and facilitators to engage in physical activity during the COVID-19 pandemic. Gamma-weighted generalized linear models and tree-structured regression models were employed to determine the associations between physical activity and health-related quality of life.

**Results:** Of 199 responses, 69% reported performing less physical activity compared to pre-pandemic. Tree-structured regression models revealed that lower leisure-time physical activity was significantly associated (*p* ≤ 0.009) with higher depression and fatigue, but lower vitality. The closure of leisure facilities and organized sport (27%) was the most commonly cited barrier to engage in physical activity, while 31% of participants mentioned concerns around their physical and mental health as a facilitator.

**Conclusion:** Our analysis identified homogenous subgroups for depression, fatigue, and vitality based specifically on leisure-time physical activity cut points, irrespective of additional demographic or situational characteristics. Findings highlight the importance of and need to safely promote leisure-time physical activity during the COVID-19 pandemic in this at-risk population to help support health-related quality of life.

## Introduction

The severe acute respiratory syndrome coronavirus 2 (SARS-CoV-2) is the causative agent of the serious life-threatening coronavirus disease of 2019 (COVID-19). COVID-19 is a respiratory infectious disease that can cause considerable damage to various bodily systems (e.g., lungs, heart, and brain) and may even lead to death (1–3). The World Health Organization (WHO) declared the COVID-19 outbreak a Public Health Emergency of International Concern on January 30, 2020 and on March 11, 2020, the outbreak was declared a global pandemic (4). Due to its rapidly increasing prevalence and high reproduction rate (i.e., the number of secondary infections generated from one infected individual) (5), unprecedented restrictions were put into place to manage the spread of the disease. For example, during the first lockdown in the United Kingdom (UK), initiated on 23rd March 2020, people were only allowed to leave their home for food supplies and to receive medical treatment, as well as once per day for exercise. Schools were closed and people were instructed to work from home. Individuals with a neurologically-related mobility disability present with a heightened prevalence of comorbidities (e.g., respiratory dysfunction, cardio- and cerebrovascular diseases, systemic immune depression, and chronic inflammation and obesity), which can predispose them to poorer outcomes (e.g., mortality and mechanical ventilation) after developing SARS-CoV-2 (3). As such, those who were deemed vulnerable due to underlying health conditions were advised to shield (i.e., not to leave their home at all).

Even though these aforementioned precautions were deemed necessary to restrict virus spread, these extreme measures have resulted in unintended consequences. There is emerging evidence that the prevalence of mental health problems has increased during the COVID-19 pandemic (6–8). This is particularly evident in those with neurological diseases and associated physical health problems (9), who are often already at increased risk for experiencing mental health issues (10).

Physical activity (PA) is a behavioral factor, which has been shown to improve mental health in the general population, as well as in individuals with neurologically-related mobility disabilities (11, 12). Indeed, higher levels of PA have been related to better mental health during the COVID-19 pandemic (13, 14). However, as a result of the restrictions to contain the spread of COVID-19, opportunities for being physically active have been limited. This is accentuated for those who might rely on exercise facilities (e.g., gyms with accessible equipment) and additional support (e.g., carers, trainers) to be physically active, such as people with a neurologically-related mobility disability. In addition, people who were advised to shield also had less opportunity to go outside for PA. Compared to non-disabled people, a greater percentage of people with a disability indicated that COVID-19 had reduced their ability to be physically active (15), with 44% indicating that they did not feel they had the opportunity to be as active as they wanted to be. This was significantly higher compared to pre-pandemic, but also compared to non-disabled individuals (15).

Given the reported impact of PA on mental health and well-being in other population groups, the aim of this study was to explore the associations between PA and health-related quality of life (HRQoL) in individuals with a neurologically-related mobility disability during COVID-19 restrictions. We utilized free-text questions to also provide context around key barriers and facilitators to performing PA or exercise during initial lockdown restrictions, thereby providing additional insight into the knowledge and practices of participants.

## Methods

### Participants and Sample Size

Owing to the exploratory nature of this study, no a-priori sample size was proposed. We sought to purposefully recruit a diverse range of participants with a neurologically-related mobility disability with the following inclusion criteria: individuals aged 18 years or over with a self-reported clinical diagnosis of a neurological condition, resulting in upper and/or lower limb mobility impairments. Ethical approval was obtained from the University of Birmingham Science, Technology, Engineering and Mathematics ethics committee (ERN_20-0689) (18/05/2020). All participants provided informed consent electronically prior to completing the e-survey.

### Participant Recruitment

Individuals were recruited through social media advertisements promoted by various charities and organizations for neurological conditions in the UK (see Acknowledgments). Prospective participants received an information sheet, provided informed consent, and completed the 25 min e-survey questionnaire between May 28th and July 25th, 2020. During this time period the following response measures were implemented in the UK: stay-at-home orders for the general population but with partially relaxed measures (ended July 4th), stay-at-home recommendations for risk groups or vulnerable populations (i.e., the elderly, people with underlying health conditions, individuals with physical disabilities) (ended July 5th), which transitioned into partially relaxed measures (16). Importantly, these data were collected before gyms and leisure facilities reopened and corresponded to a Government Response Stringency Index [GRSI: composite measure based on nine response indicators (17), rescaled to a value between 0 and 100, with 100 = strictest] of 64.4–73.2.

### Survey Development and Outcome Measures

An e-survey was created on Online Surveys (formerly BOS) (see Appendix A). The open survey was designed, and results were analyzed and reported in accordance with the Checklist for Reporting Results of Internet E-Surveys (CHERRIES) (18), see Appendix B. The survey could be completed on any electronic device with internet access. Survey structure and content were informed by a review of current evidence, including existing validated patient reported outcome measures (PROMs) and author expertise (TN, NH, SF, and JV). The survey comprised primarily closed questions with open ended questions for additional information where appropriate e.g., challenges, facilitators, and barriers. The survey was developed to capture the following:

1. general participant demographics (e.g., age, sex, and ethnicity) and current living situation to mitigate the risk of catching COVID-19 [e.g., self-imposed isolation/shielded (considered at-risk), isolation due to government legislation (e.g., working from home or furloughed), practicing social distancing, none of the above or other],
2. clinical diagnosis (neurological condition, time since diagnosis, mobility device used, and additional information where appropriate),
3. the degree of functional disability and pain, assessed via the Health Assessment Questionnaire Standardized Disability Index (HAQ-SDI) (19) and 11-point numerical rating scale (20), respectively,
4. PA and sedentary behavior, determined using the Physical Activity Scale for Individuals with Physical Disabilities (PASIPD) (21). Briefly, the main outputs from this questionnaire are energy expenditure [metabolic equivalents (METs) h/d] for leisure time physical activities (LTPA), housework and occupational activities, as well as total energy expenditure. These values are obtained by multiplying the average hours per day spent performing certain activities by a MET value indicative of the intensity of each activity. Participants were also asked how their PA levels have changed compared to pre-pandemic. Responses were recorded using a five-point Likert scale (22) to assess to what extent participants agree (“slightly more,” “considerably more”) or disagree (“slightly less, considerably less”) with the question, with a neutral option in-between (“about the same”).
5. fear of COVID-19 score, determined via the validated Fear of COVID-19 Scale (23),
6. loneliness, determined via the UCLA Loneliness Scale (24),
7. subjective vitality (eudemonic well-being), quantified via the Subjective Vitality Scale (SVS) (25),
8. symptoms of fatigue, determined via the Fatigue Severity Scale (FSS) (26),
9. the prevalence of anxious and depressive symptoms, assessed via the Hospital Anxiety and Depression Scale (HADS) (27),
10. experiences during the COVID-19 pandemic, open ended questions identifying barriers and facilitators to perform exercise or physical activity and specific challenges encountered at this moment in time.

More detailed information on the validated assessment tools and e-survey used to quantify the above outcome measures can be found in Appendix C.

### Data Preparation and Analysis

Initially, the type and pattern of missing data was assessed. Briefly, we tested the hypothesis that the missing data is missing at random (MAR) and visually explored the pattern of missing data using the R package finalfit. Visual inspection of the density- and QQ-plots as well as with the Shapiro-Wilk test of normality followed. As data was not normally distributed for any of the variables (Supplementary Table 1: density and qq-plots for all variables are located at https://github.com/jutzca/COVID-19_Excercise_Neurological_Conditions/tree/main/Figures), non-parametric tests were employed for the statistical analyses. Information related to participant demographics and neurological condition are presented with descriptive statistics [median, interquartile range (IQR), Q1, Q3, percentages].

To address the question if PA and HRQoL outcomes were associated during the COVID-19 pandemic and consequential measures, Gamma-weighted generalized linear models (GLMs) were employed. The Gamma weights were chosen to account for the skewed data distribution of the independent variables. Separate models were designed for each dependent variable, namely HAQ-SDI, fatigue, anxiety, depression, subjective vitality, pain, and change in PA. Independent variables consisted of: predominant mobility aid used, PASIPD total score and sub scores (e.g., LTPA score, household activity score, and work-related activity score), sedentary hours, GRIS, fear of COVID-19, and loneliness. Covariates included age, sex, neurological condition, and duration of condition. *Post-hoc* pairwise comparisons were Bonferroni corrected to account for multiple comparisons (28).

Additionally, we aimed to divide the initial heterogeneous patient population into successively disjoint and more homogeneous pairs of subgroups with regard to the clinical endpoint of interest. To this end, we performed an unbiased recursive partitioning technique called conditional inference tree (URP-CTREE), which is a tree-structured regression model based on sequential tests of independence between predictors and a specified clinical endpoint (29). Importantly, URP does not assume linearity, considers all possible interactions between the independent variables, handles multicollinearity, and, provides distinct cut-offs—that is, specific values of a variable that infers a given outcome (29).

Diverging stacked bar charts were used to visualize the Likert scale data. Kruksal-Walis tests were employed to test if there is a difference in distribution of the responses between sexes, neurological conditions, and mobility aid, respectively. Posteriori content analysis was used for data generated from open ended questions (challenges, barriers, and facilitators) involving two researchers (NH, JV). This resulted in additional themes/categories which were quantified with calculation of frequencies (30). Participant quotes are included to further illustrate participants free text responses to these open-ended questions (Appendix C).

R Statistical Software Version 3.5.2 for Mac Os was used for the analysis and creating the figures.

## Results

### Cohort Summary

A total of 199 individuals completed the e-survey. The cohort median age was 56.0 years (Q1–Q3: 44.0–65.0 years), 142 (71.4%) were female, and 188 (94.5%) were Caucasian whites. The most frequent neurological condition was multiple sclerosis (*n* = 67, 33.7%), followed by Parkinson's disease (*n* = 36, 18.1%), and spinal cord injury (SCI) (*n* = 32, 16.1%). Almost half of the participants reported to be in self-imposed isolation/shielding (i.e., considered at-risk) (*n* = 99, 49.7%), while the remainder were practicing social distancing (*n* = 66, 33.2%), were in isolation due to government legislation (i.e., working from home or furloughed) (*n* = 25, 12.6%), or reported none/other measure (*n* = 9, 4.5%). Detailed cohort characteristics and descriptive statistics for HRQoL outcomes are provided in Tables 1, 2, respectively.

**Table 1:** Cohort summary.

	**Overall (*N* = 199)**
**Sex**, ***n*****(%)**	
Female	142 (71.4%)
Male	56 (28.1%)
Prefer not to disclose	1 (0.5%)
**Age (years)**	
Median [Q1, Q3]	56.0 [44.0, 65.0]
**Ethnicity**, ***n*****(%)**	
Asian/Asian British	2 (1.0%)
Black/African/Caribbean/Black British	1 (0.5%)
Caucasian/White	188 (94.5%)
Mixed/multiple ethnic groups	4 (2.0%)
Other	4 (2.0%)
**Condition**	
Cerebral palsy
*n* (%)	11 (5.5%)
Median duration [Q1, Q3] (years)	32.0 [29.5, 40.2]
Fibromyalgia, chronic fatigue syndrome, CRPS
*n* (%)	15 (7.5%)
Median duration [Q1, Q3] (years)	6.25 [5.75, 15.2]
Muscular dystrophy, neuromuscular diseases
*n* (%)	22 (11.1%)
Median duration [Q1, Q3] (years)	21.3 [12.3, 30.9]
Multiple sclerosis
*n* (%)	67 (33.7%)
Median duration [Q1, Q3] (years)	13.5 [6.75, 24.5]
Parkinson's disease	
*n* (%)	36 (18.1%)
Median duration [Q1, Q3] (years)	4.75 [2.65, 8.31]
Spinal cord injury	
*n* (%)	32 (16.1%)
Median duration [Q1, Q3] (years)	9.75 [5.00, 18.0]
Other (stroke, ataxia's, spina bifida, dystonia)
*n* (%)	16 (8.0%)
Median duration [Q1, Q3] (years)	15.5 [7.00, 30.7]
**Mobility aid**, ***n*****(%)**	
Manual wheelchair	35 (17.6%)
Power wheelchair	20 (10.1%)
Mobility scooter	6 (3.0%)
Zimmer frame	12 (6.0%)
Walking sticks	43 (21.6%)
Crutches	10 (5.0%)
None	70 (35.2%)
Other	3 (1.5%)
**Situation**, ***n*****(%)**	
Self-imposed isolation/shielded (considered at-risk)	99 (49.7%)
Isolation due to government legislation	25 (12.6%)
Practicing social distancing	66 (33.2%)
None of the above	5 (2.5%)
Other	4 (2.0%)

**Table 2:** Summary of outcomes stratified by neurological conditions.

	**Cerebral Palsy (*n* = 11)**	**Fibromyalgia, Chronic fatigue syndrome, CRPS (*n* = 15)**	**Muscular dystrophy, neuromuscular diseases (*n* = 22)**	**Multiple Sclerosis (*n* = 67)**	**Parkinson's disease (*n* = 36)**	**Spinal Cord Injury (*n* = 32)**	**Other (*n* = 16)**	**Overall (*n* = 199)**
**HAQ SDI**								
Median [Q1, Q3]	1.63 [1.22, 1.75]	1.38 [1.00, 1.88]	2.00 [1.75, 2.25]	1.43 [1.00, 1.94]	0.57 [0.37, 1.14]	1.69 [1.47, 2.16]	1.81 [1.47, 2.00]	1.50 [1.00, 2.00]
**Pain**								
Median [Q1, Q3]	4.00 [2.50, 5.50]	6.00 [3.50, 8.00]	5.50 [3.25, 7.00]	3.00 [1.00, 6.00]	3.00 [1.00, 5.00]	6.00 [2.75, 7.25]	6.00 [2.00, 8.00]	4.00 [2.00, 7.00]
**Fear of COVID 19 SCORE**								
Median [Q1, Q3]	19.0 [15.0, 20.5]	18.0 [12.0, 22.5]	16.0 [14.3, 22.0]	19.0 [14.5, 22.0]	16.0 [11.8, 20.3]	17.0 [10.8, 22.3]	17.0 [14.0, 19.3]	17.0 [13.0, 22.0]
**UCLA loneliness SCORE**								
Median [Q1, Q3]	50.0 [39.0, 52.5]	47.0 [38.5, 51.0]	42.0 [30.8, 53.5]	45.0 [35.0, 56.5]	40.0 [33.5, 45.5]	42.5 [27.8, 54.3]	58.5 [49.3, 62.0]	44.0 [33.5, 55.0]
**SVS SCORE**								
Median [Q1, Q3]	3.00 [2.67, 3.42]	2.17 [1.59, 2.83]	2.67 [1.75, 3.63]	3.17 [1.67, 4.17]	3.33 [2.13, 3.87]	3.34 [2.12, 4.17]	1.83 [1.59, 2.54]	3.00 [1.67, 4.00]
**FSS SCORE**								
Median [Q1, Q3]	40.0 [30.5, 53.5]	59.0 [56.0, 62.5]	51.0 [35.5, 58.0]	50.0 [39.5, 58.0]	42.5 [31.8, 46.3]	47.0 [26.8, 62.0]	49.5 [41.8, 55.3]	48.0 [37.0, 58.0]
**Global fatigue**								
Median [Q1, Q3]	6.00 [4.00, 7.00]	4.00 [1.50, 6.50]	5.00 [3.00, 7.00]	5.00 [3.00, 8.00]	5.50 [4.00, 8.00]	6.50 [3.00, 8.00]	4.00 [2.00, 6.00]	5.00 [3.00, 8.00]
Anxiety SCORE^*^								
Median [Q1, Q3]	9.00 [6.00, 12.0]	10.0 [6.50, 14.0]	5.00 [4.00, 8.75]	7.00 [5.00, 11.0]	6.50 [5.00, 9.00]	7.50 [2.00, 11.0]	9.50 [7.25, 13.0]	7.00 [5.00, 11.0]
Normal, *n* (%)	4 (36.4%)	5 (33.3%)	13 (59.1%)	37 (55.2%)	23 (63.9%)	16 (50.0%)	4 (25.0%)	102 (51.3%)
Borderline abnormal, *n* (%)	3 (27.3%)	3 (20.0%)	6 (27.3%)	12 (17.9%)	7 (19.4%)	6 (18.8%)	5 (31.2%)	42 (21.1%)
Abnormal, *n* (%)	4 (36.4%)	7 (46.7%)	3 (13.6%)	18 (26.9%)	6 (16.7%)	10 (31.2%)	7 (43.8%)	55 (27.6%)
Depression SCORE^*^								
Median [Q1, Q3]	6.00 [4.00, 7.00]	9.00 [6.00, 10.5]	6.00 [5.00, 10.0]	8.00 [5.00, 11.0]	5.00 [3.00, 7.00]	7.00 [2.75, 11.0]	10.5 [6.50, 12.5]	7.00 [4.00, 10.0]
Normal, *n* (%)	10 (90.9%)	6 (40.0%)	13 (59.1%)	33 (49.3%)	29 (80.6%)	17 (53.1%)	5 (31.2%)	113 (56.8%)
Borderline abnormal, *n* (%)	1 (9.1%)	5 (33.3%)	4 (18.2%)	16 (23.9%)	5 (13.9%)	6 (18.8%)	3 (18.8%)	40 (20.1%)
Abnormal, *n* (%)	0 (0%)	4 (26.7%)	5 (22.7%)	18 (26.9%)	2 (5.6%)	9 (28.1%)	8 (50.0%)	46 (23.1%)

*HAQ SDI, Health Assessment Questionnaire - Standard Disability Index; FSS, Fatigue Severity Scale; SVS, Subjective Vitality Scale, UCLA, University of California Los Angeles*.

* *Scores across the respective 7 items for anxious and depressive symptoms from the Hospital Anxiety and Depression Scale (HADS) were summed together and the following scoring thresholds utilized to characterize participants: 0–7 = Normal, 8–10 = Borderline abnormal (borderline case), 11–21 = Abnormal (case)*.

### Survey Data: Response Rate and Missing Data

The response rate was excellent with only 0.1% (*n* = 12 observations across 10 variables) unanswered questions or parts thereof (Supplementary Table 2). Our analysis revealed that the missing data do have a relationship with other variables in the dataset (e.g., strenuous sport hours per day AND strenuous sport score), but the actual values that were missing are random (i.e., MAR) (Supplementary Figure 1). As a consequence, we omitted the participants in whom the variable of interests were missing for our analysis as we had sufficient power with complete cases to examine the relationships of interest.

### Physical Activity and Sedentary Behavior

**Figure 1:**
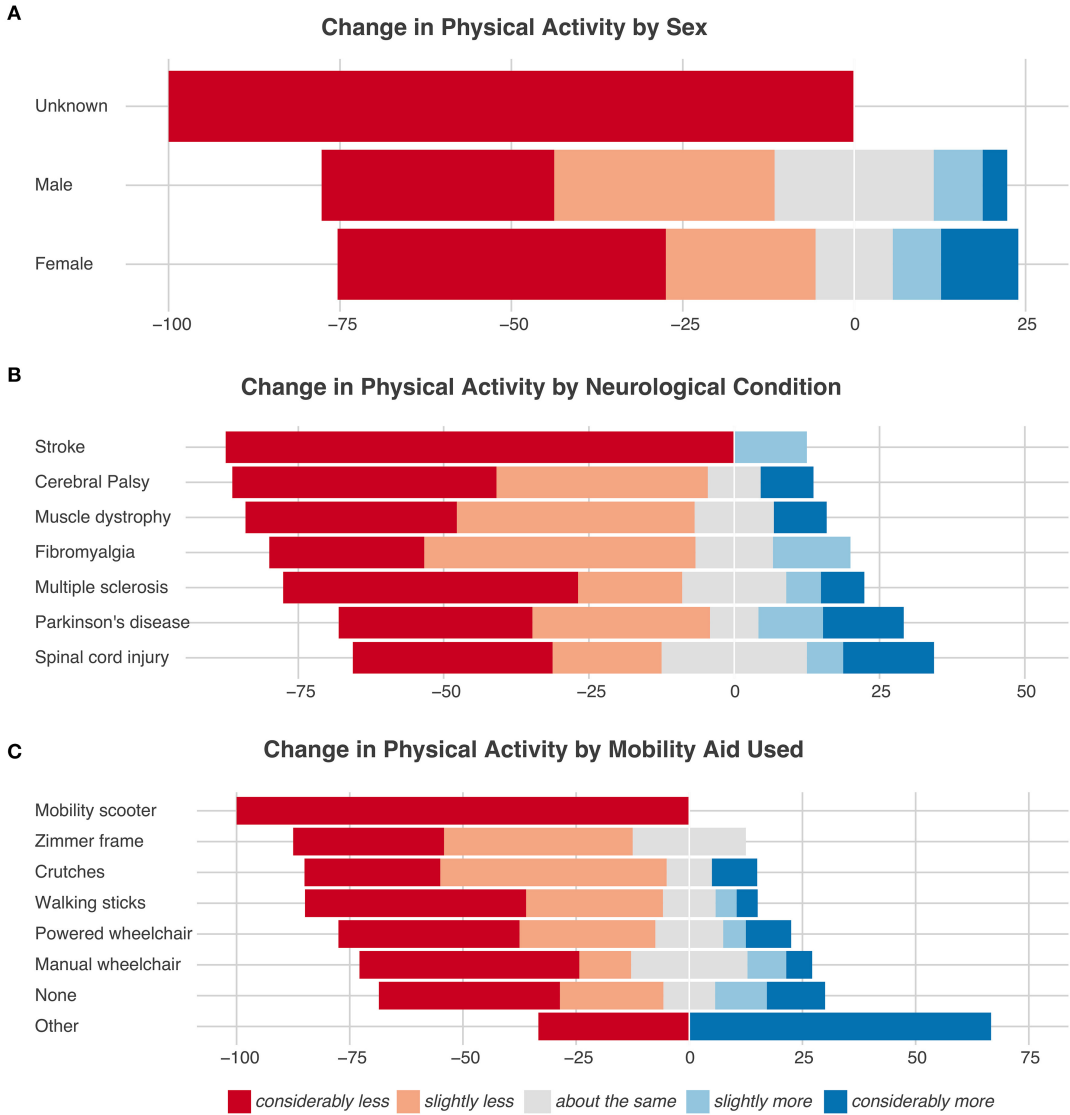
Change in physical activity stratified based on sex **(A)**, neurological condition **(B)**, and mobility aid used **(C)**.

As shown in Figure 1, 69% participants reported performing less (ranging from considerably to slightly less) PA compared to pre-COVID times and these findings were independent of sex (Figure 1A), neurological condition (Figure 1C), and mobility aid used (Figure 1C). There was no significant difference in the distribution of responses between sexes (chi-squared = 2.80, *df* = 2, *p* = 0.25), neurological conditions (chi-squared = 2.24, *df* = 6, *p* = 0.90), and mobility aid used (chi-squared = 8.22, df = 7, *p* = 0.31). The median daily time spent performing sedentary behaviors was 4.29 [Q1: 2.57, Q3: 4.29] h/day. The median daily time spent performing moderate and strenuous sports were both 0 [Q1: 0, Q3: 0]. Furthermore, participants reported to be walking/wheeling [median = 0.11 (Q1: 0, Q3: 0.75) h/day] or performing light sporting activities [median= 0.11 (Q1: 0, Q3: 0.43) h/day] for a small duration per day. Table 3 provides an overview of the hours spent per day for all the activities reported.

**Table 3:** Summary of physical activity.

	**Overall (N=199)**
**Sedentary [hours/day]**	
Median [Q1, Q3]	4.29 [2.57, 4.29]
0 h, *n* (%)	2 (1.0%)
**Walking wheeling [hours/day]**	
Median [Q1, Q3]	0.11 [0, 0.75]
0 h, *n* (%)	52 (26.1%)
**Light sport [hours/day]**	
Median [Q1, Q3]	0.11 [0, 0.43]
Missing	1 (0.5%)
0 h, *n* (%)	93 (46.7%)
**Moderate sport [hours/day]**	
Median [Q1, Q3]	0 [0, 0]
0 h, *n* (%)	162 (81.4%)
**Strenuous sport [hours/day]**	
Median [Q1, Q3]	0 [0, 0]
Missing	2 (1.0%)
0 h, *n* (%)	161 (80.9%)
**Exercise [hours/day]**	
Median [Q1, Q3]	0 [0, 0.25]
0 h, *n* (%)	116 (58.3%)
**Light housework [hours/day]**	
Median [Q1, Q3]	0.32 [0.11, 1.29]
0 h, *n* (%)	24 (12.1%)
**Heavy housework [hours/day]**	
Median [Q1, Q3]	0 [0, 0.11]
Missing	1 (0.5%)
0 h, *n* (%)	117 (58.8%)
**Home repairs [hours/day]**	
Median [Q1, Q3]	0 [0, 0]
Missing	1 (0.5%)
0 h, *n* (%)	160 (80.4%)
**Yard work [hours/day]**	
Median [Q1, Q3]	0 [0, 0.11]
0 h, *n* (%)	146 (73.4%)
**Gardening [hours/day]**	
Median [Q1, Q3]	0 [0, 0.250]
0 h, *n* (%)	103 (51.8%)
**Caring [hours/day]**	
Median [Q1, Q3]	0 [0, 0.32]
Missing	1 (0.5%)
0 h, *n* (%)	134 (67.3%)
**Work related activity [hours/day]**	
Median [Q1, Q3]	0 [0, 0]
0 h, *n* (%)	173 (86.9%)
**PASIPD SCORE [MET h/d]**	
Median [Q1, Q3]	7.18 [2.87, 14.9]
**LTPA SCORE (MET h/d)**	
Median [Q1, Q3]	2.83 [0.61, 6.43]
**Household activity SCORE, (MET h/d)**	
Median [Q1, Q3]	2.41 [0.61, 6.93]
**Work related activity SCORE, (MET h/d)**	
Median [Q1, Q3]	0 [0, 0]

*LTPA, Leisure Time Physical Activity; PASIPD, Physical Activity Scale for Individuals with Physical Disabilities*.

### Impact of COVID-19 on Physical Activity and Health-Related Quality of Life

The GLMs revealed significant associations between HAQ-SDI and the situation, neurological condition, and mobility aid used (Supplementary Table 3). Similarly, the degree of pain was associated with the neurological condition and the situation (Supplementary Table 4). Moreover, a significant relationship between fatigue and the LTPA score was found (Supplementary Table 5). No significant associations were found for depression (Supplementary Table 6), anxiety (Supplementary Table 7), loneliness (Supplementary Table 8) or vitality (Supplementary Table 9).

**Figure 2:**
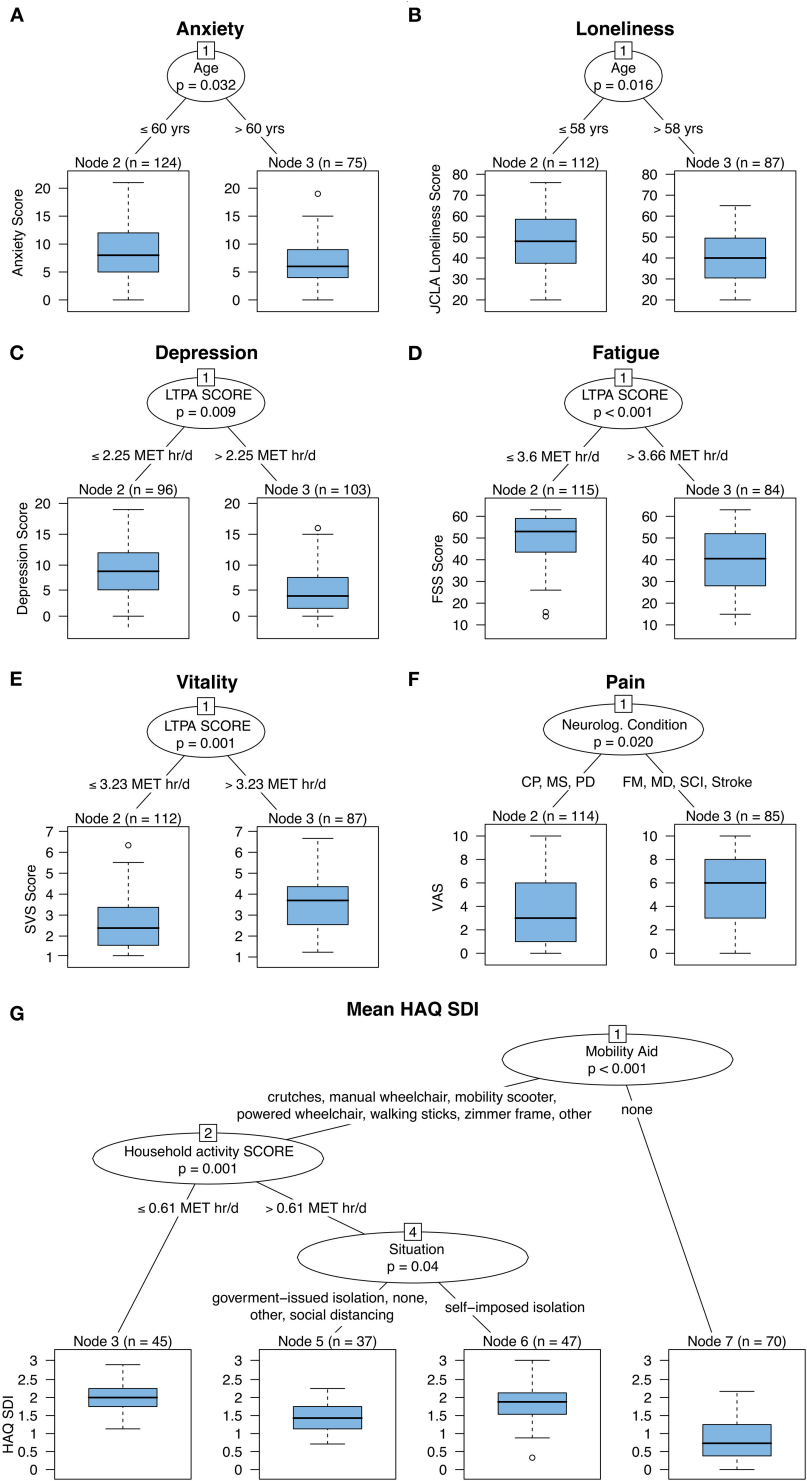
Unbiased recursive partitioning with conditional inference tree (URP-CTREE) for anxiety **(A)**, loneliness **(B)**, depression **(C)**, fatigue **(D)**, vitality **(E)**, pain **(F)**, and HAQ SDI **(G)**. The initial cohort comprises 199 participants with neurologically-related mobility disabilities. Across outcomes, LTPA score and age were the most common discriminators. CP, cerebral palsy; FM, Fibromyalgia, chronic fatigue syndrome & chronic pain syndrome; FSS, fatigue severity scale; HAQ SDI, health assessment questionnaire standardized disability index; LTPA, leisure time physical activity; MD, muscular dystrophy & neuromuscular diseases; MET, metabolic equivalents; MS, multiple sclerosis; PD, Parkinson's disease; SCI, spinal cord injury; Stroke (other, ataxia's, spina bifida, dystonia); SVS, subjective vitality scale; VAS, visual analog scale.

**Figure 3:**
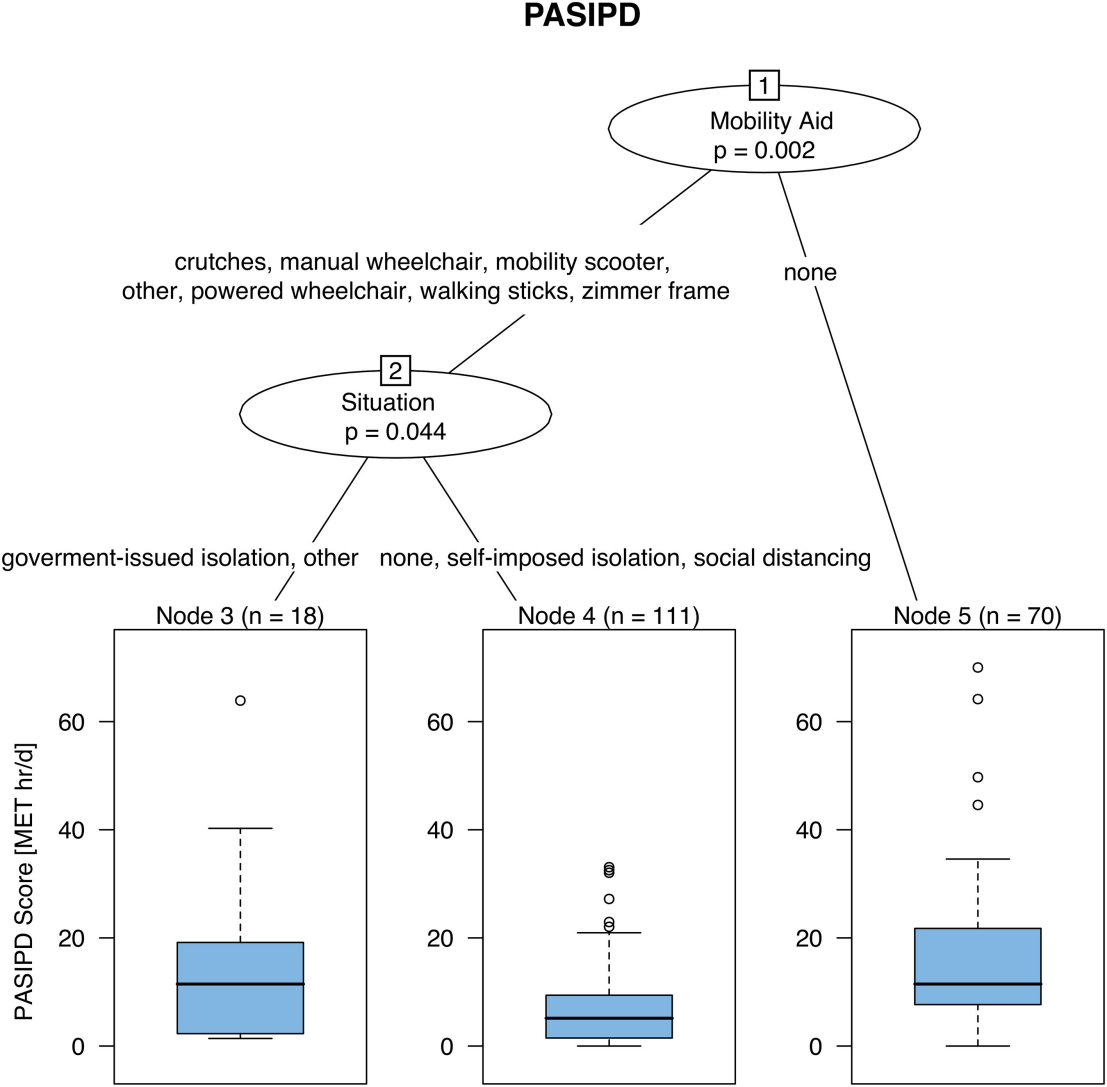
Unbiased recursive partitioning with conditional inference tree (URP-CTREE) for total physical activity in 199 participants with neurologically-related mobility disabilities. Participants requiring no walking aid reported the highest PASIDP score, while patients relying on any kind of mobility aid AND self-isolating/social distancing were found to have the lowest PASIDP score. MET, metabolic equivalents; PASIPD, physical activity scale for individuals with physical disabilities.

Taking into account interactions between variables, the URP-CTREE illustrates that younger participants (<60 years of age) reported higher anxiety scores (Figure 2A), while older participants (≥58 years of age) felt lonelier (Figure 2B). Moreover, higher depression and fatigue scores were associated with lower LTPA scores [depression: cut off: ≤ 2.25 MET h/d (Figure 2C); fatigue: cut off: ≤ 3.66 MET h/d (Figure 2D)]. In contrast, higher vitality scores were associated with higher LTPA scores (cut off: <3.26 MET h/d, Figure 2E). Higher pain scores (mean = 5.26, *n* = 85) were reported by participants with fibromyalgia, muscular dystrophy, stroke, or SCI compared to those with cerebral palsy, multiple sclerosis, or Parkinson's disease (mean = 3.45, *n* = 114; Figure 2F). Moreover, the HAQ-SDI score was the lowest for participants without mobility aids and the highest for participants using any mobility aid AND a household activity score of ≤ 0.61 MET h/d (Figure 2G). Lastly, the PASIPD score was highest for participants who were not using any mobility aids (mean = 2.05 MET h/d, *n* = 45). Participants who used any kind of mobility aid AND had no restriction, self-imposed isolation (i.e., considered at risk), or social distancing reported the lowest PASIPD score (mean = 1.44 MET h/d, *n* = 37) (Figure 3).

### Challenges, Barriers, and Facilitators: Results From Free Text Data

The response rates to the optional free text questions related to challenges, barriers, and facilitators were high with 99.0% (197/199), 98.5% (196/199), and 96.5% (192/199), respectively. Almost half the participants (n=91/197) reported a lack of “normal life/lockdown” as a challenge during this period, with around a quarter missing or not seeing family (*n* = 44/197). Additionally, “fear/uncertainty” or “isolation” were reported as a challenge by 30 participants.

“Closed gyms/pool/organized sport” was the most commonly reported barrier to engage in PA by participants (*n* = 53/196), with a “lack of motivation” being second (*n* = 30/196). Similar frequencies were reported for barriers relating to “fatigue” (*n* = 25/196), “Leaving home” (*n* = 24/196), “Lack of equipment/space/support” (*n* = 24/196) and “Fear/including hurting/pain” (*n* = 22/196).

In terms of facilitators to engage in PA, almost a third of participants reported factors associated with well-being to be facilitating; “Health (mental and physical)/weight” (*n* = 60/192). Beyond this, similar frequencies were evident for “Nothing/same as ever” (*n* = 34/192), “Family/Healthcare practitioner support” (*n* = 30/192), “Online classes” (*n* = 28/192), and “Leaving the house/fresh air/garden” (*n* = 27/192).

Representative participant quotes to illustrate themes/categories derived from the open-ended questions can be found in Appendix C.

## Discussion

This study demonstrates the detrimental impact of the COVID-19 pandemic and ensuing restrictions on PA behaviors in a heterogeneous sample of adults with a neurologically-related mobility disability living in the UK. Our findings attempt to address a key research gap identified by a recent review (31), namely the lack of early research investigating the impact of COVID-19 on individuals with a physical disability. Using a comprehensive battery of valid and reliable PROMs we revealed that LTPA was significantly associated with HRQoL outcomes. Specifically, higher levels of LTPA were related to lower depression and fatigue scores, as well as higher subjective vitality. The closure of leisure facilities and lack of motivation were deemed key barriers to engage in PA/exercise, while concerns around health (both physical and mental) was reported as a key facilitator by approximately a third of participants. Collectively these free text data provided additional insight into the knowledge and experiences of this population during the COVID-19 pandemic.

### Self-Reported Physical Activity Behaviors

A scoping review indicated that PA levels in the general population were reduced during the pandemic (32). This is perhaps unsurprising given gyms and swimming pools were closed, and sports or other exercise classes were all stopped. Consequently, the only opportunities for being active were home-based exercise or outdoor PA in the local area, such as walking or cycling. However, individuals with a neurologically-related mobility disability may represent the archetypal patient population of concern during the COVID-19 pandemic. Even before the introduction of government legislation to tackle this pandemic, these individuals reported low levels of PA and multiple obstacles to perform exercise, despite the interest to do so (33, 34). Reported barriers include a perceived low return on physical investment, lack of accessible facilities, unaffordable equipment, no personal assistance and fears regarding safety and injury (35, 36). Such environmental factors were noted in participants responses to open ended questions (Appendix C). Seemingly, COVID-19 restrictions have magnified the environmental and personal barriers commonly experienced by individuals with a disability to perform PA.

Worryingly, sixty-nine percent of our middle-aged cohort reported performing less PA compared to pre-pandemic. Cross-sectional data (*n* = 125) collected during the same time frame in the UK reported that 61% of children and young adults with physical and/or intellectual disabilities were less physically active as a result of lockdown restrictions (37). Outside of the UK, decreased PA was observed in 44% of Parkinson's disease patients in Japan (data collected over a wider timeframe between June and December 2020) (38). The COVID-19 quarantine in Italy significantly decreased total weekly PA levels (quantified via the International Physical Activity Questionnaire Short-Form before and during) in a sample of participants with neuromuscular disease (*n* = 149). The average total PASIPD score (median: 7.18 MET h/day) reported in this current study is less than half that of previous studies (19.40–20.50 MET h/day), which assessed middle-aged individuals with neurological conditions and a similar disability severity outside of a global pandemic (21, 39, 40). A similar total PASIPD score (mean: 7.95 ± 7.91 MET h/day) was reported during lockdown in Spain for twenty individuals with motor-complete SCI (reduced from 26.36 ± 19.09 MET hr/day pre-lockdown) (41). This reduction was mainly explained by a substantial reduction in the LTPA sub-score. Consequently, our results and others highlight that the low levels of PA commonly reported in this population have been further exacerbated by strict lockdown protocols and the closure of non-essential support services.

To provide further context, between 58.3 and 81.0% of our participants reported zero h/day for performing activities above the intensity threshold necessary to improve fitness/health (Table 3: moderate or strenuous sport and recreation, exercise for strength and endurance, heavy housework). These data imply that during the COVID-19 pandemic, the majority of individuals with a neurologically-related mobility disability were unable to achieve volumes of moderate-intensity PA (>150 min per week) sufficient to promote substantial health benefits for disabled adults (42). When investigating factors that were linked with the total PASIPD score reported at this time (Figure 3), the URP-CTREE split was initially for mobility aid usage followed by COVID-19 living situation. Consequently, participants who used any kind of mobility aid and considered themselves at-risk (i.e., self-imposed isolation/shielded), no restriction or practicing social distancing reported the lowest total PASIPD score. It is therefore intuitive to propose that individuals with the greatest mobility impairment or disability severity and greatest perceived vulnerability, irrespective of other factors (e.g., age, specific neurological condition, sex), require extra support to perform PA during this challenging time.

### Physical Functioning and Health-Related Quality of Life

The median HAQ-SDI scores of our cohort (1.5) indicate the presence of disability (>1). Perhaps unsurprisingly, the HAQ-SDI score was lowest for participants without mobility aids but highest for individuals who used any mobility aid and performed less household PA. A subjective worsening of neurological symptoms has been reported in individuals with Parkinson's disease (38, 43) amyotrophic lateral sclerosis (ALS) (44) and cerebellar ataxias (45) as a result of COVID-19 restrictions, increasing the socio-economic burden of these neurological conditions (46). Indeed, the restricted access to healthcare services (rehabilitation, community and home-based support) during the pandemic (31) for disabled individuals may detrimentally impact mobility and function. In ALS patients during the pandemic, a greater mobility impairment and rehabilitation therapy suspension were significant predictors of anxiety symptom severity (44). Thirty-one percent of participants cited concerns around “Health (mental and physical)/weight” as key motivators/facilitators to be physically active, whereas, others voiced concerns about their rapidly declining physical status (“I have declined physically quite rapidly and the exercises I could do at home at the beginning of the lockdown are now impossible” P101). This is particularly worrisome as individuals with neuromuscular disabilities were already predisposed to severe deconditioning (e.g., reduced strength and fitness) and significant health risks (e.g., increased sarcopenia, obesity, and cardiometabolic disease risk factors) due to low rates of PA (35). It is apparent that COVID-19 confinement strategies can further compound these aforementioned health risks (47, 48) and the presence of these comorbidities may also increase the risk of poorer outcomes after developing COVID-19 (3). The pandemic-related declining fitness and functional status should be closely considered by practitioners when resuming rehabilitation and exercise interventions in individuals with neurological conditions.

### Psychological Well-Being

A survey from Activity Alliance showed that compared to non-disabled people, people with a physical disability were significantly more likely to be anxious, feel lonely, be less happy and generally more negative about the future during COVID-19 (15). Different factors have been shown to contribute to negative mental health, such as reduced social interactions, concerns about contracting the disease, as well as concerns about not being able to access appropriate healthcare when needed (49, 50). Indeed, participants reported missing or not seeing family (22%) and “fear/uncertainty” or “isolation” (both 15%) as being especially challenging during the COVID-19 pandemic. Despite being considered a vulnerable cohort, the fear of COVID-19 score was not higher than those reported in the general population (17 vs. 15.6–18.3) (51). Our analyses also indicated fear of COVID-19 score was not associated with any HRQoL outcomes. In line with previous reports, our analysis uncovered that younger participants reported higher anxiety scores while older participants felt lonelier (52, 53). It is possible that older adults were less affected by personal and emotional problems during this time (i.e., perhaps retired, therefore less concerned about job security and financial worries). The increased loneliness may be due to older adults shielding for a longer period of time and therefore not experiencing direct contact with family members or friends. Additionally, older adults may be less likely to successfully utilize digital technology (such as online social media and video chat platforms), which may increase social connectedness and mitigate loneliness (54).

A systematic review unequivocally stated that the COVID-19 pandemic and ensuing containment strategies has caused psychological distress (55). However, conflicting data suggest no worsening in symptoms of anxiety or depression in individuals with a neurological condition as a result of COVID-19 restrictions (56, 57), implying a level of resilience. Our findings indicated that 27 and 23% of our cohort were experiencing abnormal symptoms of anxiety and depression, respectively. Comparative cross-sectional HADS data, collected between July to September 2020 among individuals with cerebellar ataxia in Cuba, demonstrated a similar incidence of anxiety (21%) and depression (23%) (45). These rates of anxiety (17%) and depression (20%) are not too dissimilar to those reported prior to the COVID-19 pandemic for 253 individuals with multiple sclerosis (58). While this is encouraging, studies in patients with spinocerebellar and cerebellar ataxia have implied greater levels of anxiety and depression relative to controls during the COVID-19 pandemic (45, 59). Therefore, cultivating healthy coping strategies and resilience during periods of uncertainty in individuals with neurological conditions are essential to improve psychological well-being.

### Associations Between Leisure-Time Physical Activity and Psychological Well-Being

It has been argued that the aforementioned changes in HRQoL are driven by social isolation, considerable lifestyle alterations and financial and occupational health concerns triggered by the pandemic (55). One notable lifestyle alteration and potential coping strategy that we have shown to be detrimentally impacted in our cohort due to COVID-19 restrictions is PA. Indeed, multiple studies have identified associations between PA and mental health during the COVID-19 pandemic (13, 14, 60). Structural equation modeling in a mixed sample (multiple sclerosis, n = 497 and controls, *n* = 348) of Italian adults during the COVID-19 lockdown revealed exercise is a valuable tool in managing depressive symptoms (9). We demonstrate evidence that LTPA is associated with symptoms of depression, fatigue and subjective vitality in individuals with a neurologically-related mobility disability. These findings extend the associations of PA beyond mental health pathologies (i.e., depression) to other holistic psychological well-being outcomes (subjective vitality and fatigue) that are often ignored. Subjective vitality is a measure of eudaimonic well-being and is a positive psychological state that has implications for optimal functioning (25). Fatigue is commonly reported by individuals with neurological conditions and it has a substantial detrimental impact on HRQoL (61). Interestingly, homogeneous subgroups were defined with better HRQoL outcomes corresponding to achieving a relatively small LTPA energy expenditure (depression >2.25 MET h/d, fatigue: >3.66 MET h/d, vitality: >3.26 MET h/d). Individuals with physical disabilities should be encouraged to perform these corresponding volumes of LTPA to support HRQoL during the pandemic. However, the causative impact of these LTPA recommendations on HRQoL in this population remains to be longitudinally tested.

### Implications for Practice and Further Research

In the face of continued COVID-19 restrictions, or future crises, policy makers should consider the provision of services for adults with a physical disability to address exacerbated health inequalities and minimize the barriers to perform PA. Given the importance of PA for both physical and mental health, it has been argued that public health initiatives for clinical populations should incorporate the creation and implementation of interventions to promote safe PA should COVID-19 infection rates rise, prompting further lockdowns (62). To ensure enhanced feasibility and adherence, such interventions should be informed through the lived experiences of the target population. Accordingly, the contextual information gleaned from the free text questions has facilitated the recommendations described in Table 4 to best facilitate PA in individuals with neurological conditions during a global pandemic. Inclusive online exercise resources for practitioners and individuals with neurological conditions can be found at the end of Appendix C. Practitioners working with this population need to be prepared to adapt (e.g., provide home-based online exercise classes) to ensure the imposed COVID-19 restrictions do not have a persisting detrimental effect on HRQoL in individuals with neurological conditions. Longitudinal follow-up studies are warranted to understand the longer-term consequences of COVID-19 and associated containment strategies on PA behaviors and HRQoL in clinically vulnerable individuals with a neurologically-related mobility disability.

**Table 4:** Recommendations for promoting physical activity in individuals with a neurologically-related mobility disability during the COVID-19 pandemic.

**1. Consider synchronous online sessions, where the practitioner can provide real-time feedback and adapt the exercise to meet the individuals needs**
**2. Enlist help from somebody within the support bubble who is physically present, helping ensure an element of safety**
**3. The provision of cheap and inclusive exercise equipment if possible (e.g., TheraBands^®^)**
**4. Develop a vibrant online community for support and accountability amongst peers**
**5. Work in multidisciplinary teams to address the interplay with mental health considerations**

### Strengths and Limitations

These findings should be considered relative to the study's methodological strengths and limitations. Most importantly, causality cannot be inferred based on our results owing to the cross-sectional nature of the study. Due to the rapidly evolving government restrictions, the survey was not piloted among individuals with a neurological condition prior to its release. Despite this, we utilized population validated PROMs (see Appendix C). Strengths of this study include the use of a data-driven statistical approach (URP-CTREE), multidisciplinary research team and the high response rate (96.5–99.0%) to the optional free-text questions, affording greater individual insights into participants experiences during this time. The pandemic-related nocebo effect (63) may be exacerbated in individuals with neurological conditions, who were considered “at-risk” relative to the general population. The negative expectations of these individuals, possibly fueled by alarming media reports, could have amplified the discomfort and anxiety reported during the COVID-19 pandemic, above and beyond what was actually experienced. There are inherent limitations with using self-report measures to quantify PA, such as potential recall bias (64). However, using the PASIPD is advantageous as it allows the comparison of PA across a heterogenous cohort with varying degrees of mobility impairment (i.e., questions are framed for wheelchair users and ambulatory individuals). While the absolute accuracy of the assigned MET multipliers used in this instrument have not been supported (21), they at least serve as logical constants to rank order the intensity of PA. Furthermore, the PASIPD demonstrates a degree of test-retest reliability and criterion validity that is comparable to self-report PA questionnaires commonly used in the general population (65). Akin to other open e-survey research, the validity of participants self-assessment of eligibility could be deemed a limitation. Cognitive impairment, which may have impacted self-report responses, was not included as an eligibility criterion. Other notable limitations include the heterogeneity of neurological conditions in the cohort, the relatively small number of participants in each diagnostic group and possible self-selection bias, which may influence the representativeness of our cohort. Respondents were predominantly white females, which also limits the generalizability to the wider population of individuals living with a neurological condition. COVID-19 has been shown to have a disproportionate impact on ethnic minority groups (66), indicating that further research is required to address this gap.

### Conclusion

Our findings indicate that LTPA was associated with depressive symptoms, fatigue, and subjective vitality in individuals with a neurologically-related mobility disability during the COVID-19 pandemic. Understanding ways to better support individuals with a physical disability to maintain health promoting behaviors during a period of uncertainty, such as a global pandemic, war or natural disaster is of utmost importance. Further research is required to inform wider public health recommendations targeting the specific and unique needs of adults with a physical disability as COVID-19 restrictions are eased.

## Code Availability

The code to run the analysis as well as create the figures and tables can be found on our Github repository (https://github.com/jutzca/COVID-19_Excercise_Neurological_Conditions).

## Data Availability Statement

The raw data supporting the conclusions of this article will be made available by the authors, without undue reservation.

## Ethics Statement

Ethical approval was obtained from the University of Birmingham Science, Technology, Engineering and Mathematics ethics committee (ERN_20-0689). The participants provided their written informed consent to participate in this study.

## Author Contributions

Material preparation and data collection was performed by TN. Statistical analyses were performed by CJ (quantitative data), NH, and JV (qualitative data). The first draft of the manuscript was written by TN, CJ, NH, and JV. All authors contributed to the study conception and design, commented on previous versions of the manuscript, and have read and approved the final manuscript.

## Conflict of Interest

The authors declare that the research was conducted in the absence of any commercial or financial relationships that could be construed as a potential conflict of interest.

## Publisher's Note

All claims expressed in this article are solely those of the authors and do not necessarily represent those of their affiliated organizations, or those of the publisher, the editors and the reviewers. Any product that may be evaluated in this article, or claim that may be made by its manufacturer, is not guaranteed or endorsed by the publisher.

## Supplementary Material

Supplementary Figure 1 Pattern of missing data. Pink squares indicate missing values and blue squares denote complete data.
