# Supplementary material for: Physical Activity and Health-Related Quality of Life in Adults With a Neurologically-Related Mobility Disability During the COVID-19 Pandemic: An Exploratory Analysis
Source: Front Neurol. 2021 Aug 27;12:699884. doi: 10.3389/fneur.2021.699884 (PMC8429606; doi:10.3389/fneur.2021.699884)
Supplement: Supplementary file 2 [file Table_1.docx]

| **Supplementary Table 1. Results of the Shapiro-Wilk test of normality.** | | |
| --- | --- | --- |
| **Variable name** | **W-Statistic** | **P -value** |
| Anxiety SCORE | 0.975 | 0.001 |
| Caring Hours Per Day | 0.540 | 0.000 |
| Caring SCORE | 0.540 | 0.000 |
| Depression SCORE | 0.969 | 0.000 |
| Exercise Hours Per Day | 0.537 | 0.000 |
| Exercise SCORE | 0.537 | 0.000 |
| Fear of COVID 19 SCORE | 0.978 | 0.003 |
| FSS SCORE | 0.925 | 0.000 |
| Gardening Hours Per Day | 0.493 | 0.000 |
| Gardening SCORE | 0.493 | 0.000 |
| Global Fatigue | 0.958 | 0.000 |
| GRSI | 0.705 | 0.000 |
| HAQ SDI Mean | 0.976 | 0.002 |
| Heavy housework Hours Per Day | 0.556 | 0.000 |
| Heavy housework SCORE | 0.556 | 0.000 |
| Home repairs Hours Per Day | 0.252 | 0.000 |
| Home repairs SCORE | 0.252 | 0.000 |
| Household activity SCORE | 0.686 | 0.000 |
| Leaving the house to work Hours Per Day | 0.229 | 0.000 |
| Light housework Hours Per Day | 0.757 | 0.000 |
| Light housework SCORE | 0.757 | 0.000 |
| Light sport Hours Per Day | 0.638 | 0.000 |
| Light sport SCORE | 0.638 | 0.000 |
| LTPA SCORE | 0.647 | 0.000 |
| Moderate sport Hours Per Day | 0.347 | 0.000 |
| Moderate sport SCORE | 0.347 | 0.000 |
| Pain | 0.933 | 0.000 |
| PASIPD SCORE | 0.784 | 0.000 |
| Sedentary Hrs Per Day | 0.771 | 0.000 |
| Strenuous sport Hours Per Day | 0.350 | 0.000 |
| Strenuous sport SCORE | 0.350 | 0.000 |
| SVS SCORE | 0.959 | 0.000 |
| UCLA Loneliness SCORE | 0.976 | 0.002 |
| Walking wheeling Hours Per Day | 0.667 | 0.000 |
| Walking wheeling SCORE | 0.667 | 0.000 |
| Work related activity Hours Per Day | 0.317 | 0.000 |
| Work related activity SCORE | 0.317 | 0.000 |
| Yard work Hours Per Day | 0.373 | 0.000 |
| Yard work SCORE | 0.373 | 0.000 |

*Abbreviations: FSS = Fatigue Severity Scale; GRSI = Government Response Stringency Index; HAQ Index = Healthcare Access Quality Index; SDI = Socio-demographic Index; LTPA = Leisure-Time Physical activity; PASIPD = Physical Activity Scale for Individuals with Physical Disabilities; SVS = Subjective Vitality Scale; UCLA = University of California, Los Angeles;*
