# Supplementary material for: Physical Activity and Health-Related Quality of Life in Adults With a Neurologically-Related Mobility Disability During the COVID-19 Pandemic: An Exploratory Analysis
Source: Front Neurol. 2021 Aug 27;12:699884. doi: 10.3389/fneur.2021.699884 (PMC8429606; doi:10.3389/fneur.2021.699884)
Supplement: Supplementary file 3 [file Table_2.docx]

| **Supplementary Table 2. Number of missing values (NA) per variable.** | |
| --- | --- |
| **Variable name** | **Number of NA’s** |
| Unique ID | 0 |
| Age | 0 |
| Sex | 0 |
| Situation | 0 |
| Ethnicity | 0 |
| Condition | 0 |
| Duration Corrected | 0 |
| Mobility Aid | 0 |
| HAQ SDI Mean | 0 |
| Pain | 0 |
| Sedentary Hrs Per Day | 0 |
| Walking wheeling Hours Per Day | 0 |
| Walking wheeling SCORE | 0 |
| Light sport Hours Per Day | 1 |
| Light sport SCORE | 1 |
| Moderate sport Hours Per Day | 0 |
| Moderate sport SCORE | 0 |
| Strenuous sport Hours Per Day | 2 |
| Strenuous sport SCORE | 2 |
| Exercise Hours Per Day | 0 |
| Exercise SCORE | 0 |
| LTPA SCORE | 0 |
| Light housework Hours Per Day | 0 |
| Light housework SCORE | 0 |
| Heavy housework Hours Per Day | 1 |
| Heavy housework SCORE | 1 |
| Home repairs Hours Per Day | 1 |
| Home repairs SCORE | 1 |
| Yard work Hours Per Day | 0 |
| Yard work SCORE | 0 |
| Gardening Hours Per Day | 0 |
| Gardening SCORE | 0 |
| Caring Hours Per Day | 1 |
| Caring SCORE | 1 |
| Household activity SCORE | 0 |
| Work related activity Hours Per Day | 0 |
| Work related activity SCORE | 0 |
| PASIPD SCORE | 0 |
| Leaving the house to work Hours Per Day | 0 |
| Change in Physical Activity | 0 |
| Fear of COVID 19 SCORE | 0 |
| UCLA Loneliness SCORE | 0 |
| SVS SCORE | 0 |
| FSS SCORE | 0 |
| Global Fatigue | 0 |
| Anxiety SCORE | 0 |
| Depression SCORE | 0 |
| Completion Date | 0 |
| GRSI | 0 |

*Abbreviations: FSS = Fatigue Severity Scale; GRSI = Government Response Stringency Index; HAQ Index = Healthcare Access Quality Index; SDI = Socio-demographic Index; LTPA = Leisure-Time Physical activity; PASIPD = Physical Activity Scale for Individuals with Physical Disabilities; SVS = Subjective Vitality Scale; UCLA = University of California, Los Angeles;*
