# Supplementary material for: Physical Activity and Health-Related Quality of Life in Adults With a Neurologically-Related Mobility Disability During the COVID-19 Pandemic: An Exploratory Analysis
Source: Front Neurol. 2021 Aug 27;12:699884. doi: 10.3389/fneur.2021.699884 (PMC8429606; doi:10.3389/fneur.2021.699884)
Supplement: Supplementary file 4 [file Table_3.docx]

| **Supplementary Table 3. Summary statistics of GLM: HAQ SDI** | | | |
| --- | --- | --- | --- |
|  | **HAQ SDI** | | |
| *Predictors* | *Estimates* | *CI* | *p* |
| (Intercept) | 1.90 | 1.51 – 2.43 | **<0.001** |
| Age | 1.00 | 1.00 – 1.00 | 0.352 |
| Sex [male]^1^ | 1.02 | 0.94 – 1.11 | 0.615 |
| Sex [unknown]^1^ | 1.00 | 0.69 – 1.72 | 0.995 |
| Situation [none]^2^ | 1.11 | 0.79 – 1.66 | 0.579 |
| Situation [other]^2^ | 0.87 | 0.66 – 1.21 | 0.364 |
| Situation [self-imposed isolation]^2^ | 0.81 | 0.70 – 0.94 | **0.006** |
| Situation [social distancing]^2^ | 0.89 | 0.76 – 1.04 | 0.166 |
| Condition [Fibromyalgia]^3^ | 1.01 | 0.82 – 1.24 | 0.933 |
| Condition [Muscle Dystrophy]^3^ | 1.05 | 0.86 – 1.26 | 0.647 |
| Condition [Multiple Sclerosis]^3^ | 1.11 | 0.92 – 1.32 | 0.242 |
| Condition [Parkinson’s Disease]^3^ | 1.41 | 1.10 – 1.81 | **0.008** |
| Condition [Spinal Cord Injury]^3^ | 1.06 | 0.88 – 1.26 | 0.529 |
| Condition [Stroke]^3^ | 1.03 | 0.85 – 1.25 | 0.744 |
| Mobility Aid [Manual wheelchair]^4^ | 0.98 | 0.83 – 1.15 | 0.838 |
| Mobility Aid [Mobility scooter]^4^ | 1.07 | 0.84 – 1.39 | 0.585 |
| Mobility Aid [None]^4^ | 1.42 | 1.16 – 1.73 | **0.001** |
| Mobility Aid [Other]^4^ | 0.94 | 0.74 – 1.25 | 0.659 |
| Mobility Aid [Powered wheelchair]^4^ | 0.90 | 0.76 – 1.06 | 0.217 |
| Mobility Aid [Walking sticks]^4^ | 1.05 | 0.89 – 1.23 | 0.542 |
| Mobility Aid [Zimmer frame]^4^ | 0.97 | 0.79 – 1.17 | 0.732 |
| LTPA SCORE | 1.01 | 1.00 – 1.01 | 0.053 |
| Household activity SCORE | 1.01 | 1.00 – 1.02 | 0.097 |
| Work related activity SCORE | 1.00 | 0.98 – 1.02 | 0.907 |
| Sedentary Hours PerDay | 1.01 | 0.98 – 1.05 | 0.436 |
| Observations | 192 | | |
| R^2^ Nagelkerke | 0.507 | | |

*Abbreviations: LTPA = Leisure-time physical activity; HAQ Index = Healthcare Access Quality Index; SDI = Socio-demographic Index*

^1^*Reference: Female*

^2^*Reference: Government-issued isolation*

^3^*Reference: Cerebral Palsy*

^4^*Reference: Crutches*
