# Supplementary material for: Physical Activity and Health-Related Quality of Life in Adults With a Neurologically-Related Mobility Disability During the COVID-19 Pandemic: An Exploratory Analysis
Source: Front Neurol. 2021 Aug 27;12:699884. doi: 10.3389/fneur.2021.699884 (PMC8429606; doi:10.3389/fneur.2021.699884)
Supplement: Supplementary file 6 [file Table_5.docx]

| **Supplementary Table 5. Summary statistics of GLM: Fatigue** | | | |
| --- | --- | --- | --- |
|  | **FSS SCORE** | | |
| *Predictors* | *Estimates* | *CI* | *p* |
| (Intercept) | 1.02 | 1.02 – 1.03 | **<0.001** |
| Age | 1.00 | 1.00 – 1.00 | 0.374 |
| Sex [male]^1^ | 1.00 | 1.00 – 1.00 | 0.136 |
| Sex [unknown]^1^ | 0.99 | 0.98 – 1.01 | 0.271 |
| Situation [none]^2^ | 1.00 | 0.99 – 1.01 | 0.934 |
| Situation [other]^2^ | 1.00 | 0.99 – 1.00 | 0.330 |
| Situation [self-imposed isolation]^2^ | 1.00 | 1.00 – 1.00 | 0.704 |
| Situation [social distancing]^2^ | 1.00 | 1.00 – 1.00 | 0.746 |
| Condition [Fibromyalgia]^3^ | 1.00 | 0.99 – 1.00 | 0.184 |
| Condition [Muscle Dystrophy]^3^ | 1.00 | 1.00 – 1.01 | 0.573 |
| Condition [Multiple Sclerosis]^3^ | 1.00 | 0.99 – 1.00 | 0.648 |
| Condition [Parkinson’s Disease]^3^ | 1.00 | 1.00 – 1.01 | 0.500 |
| Condition [Spinal Cord Injury]^3^ | 1.00 | 0.99 – 1.00 | 0.664 |
| Condition [Stroke]^3^ | 1.00 | 0.99 – 1.01 | 0.882 |
| Mobility Aid [Manual wheelchair]^4^ | 1.00 | 1.00 – 1.01 | 0.213 |
| Mobility Aid [Mobility scooter]^4^ | 1.00 | 0.99 – 1.01 | 0.960 |
| Mobility Aid [None]^4^ | 1.00 | 0.99 – 1.00 | 0.952 |
| Mobility Aid [Other]^4^ | 1.00 | 0.99 – 1.00 | 0.222 |
| Mobility Aid [Powered wheelchair]^4^ | 1.00 | 0.99 – 1.00 | 0.690 |
| Mobility Aid [Walking sticks]^4^ | 1.00 | 1.00 – 1.00 | 0.962 |
| Mobility Aid [Zimmer frame]^4^ | 1.00 | 1.00 – 1.01 | 0.272 |
| LTPA SCORE | 1.00 | 1.00 – 1.00 | **<0.001** |
| Household activity SCORE | 1.00 | 1.00 – 1.00 | 0.636 |
| Work related activity SCORE | 1.00 | 1.00 – 1.00 | 0.282 |
| Sedentary Hours PerDay | 1.00 | 1.00 – 1.00 | 0.408 |
| Observations | 199 | | |
| R^2^ Nagelkerke | 0.205 | | |

*Abbreviations: LTPA = Leisure-time physical activity; FSS = Fatigue severity scale;*

^1^*Reference: Female*

^2^*Reference: Government-issued isolation*

^3^*Reference: Cerebral Palsy*

^4^*Reference: Crutches*
