# Supplementary material for: Physical Activity and Health-Related Quality of Life in Adults With a Neurologically-Related Mobility Disability During the COVID-19 Pandemic: An Exploratory Analysis
Source: Front Neurol. 2021 Aug 27;12:699884. doi: 10.3389/fneur.2021.699884 (PMC8429606; doi:10.3389/fneur.2021.699884)
Supplement: Supplementary file 7 [file Table_6.docx]

| **Supplementary Table 6. Summary statistics of GLM: Depression Score** | | | |
| --- | --- | --- | --- |
|  | **Depression SCORE** | | |
| *Predictors* | *Estimates* | *CI* | *p* |
| (Intercept) | 1.16 | 1.07 – 1.25 | **<0.001** |
| Age | 1.00 | 1.00 – 1.00 | 0.168 |
| Sex [male]^1^ | 1.01 | 0.98 – 1.04 | 0.436 |
| Sex [unknown]^1^ | 0.98 | 0.91 – 1.12 | 0.759 |
| Situation [none]^2^ | 0.95 | 0.89 – 1.02 | 0.150 |
| Situation [other]^2^ | 0.95 | 0.88 – 1.03 | 0.162 |
| Situation [self-imposed isolation]^2^ | 0.97 | 0.93 – 1.01 | 0.114 |
| Situation [social distancing]^2^ | 0.98 | 0.94 – 1.02 | 0.381 |
| Condition [Fibromyalgia]^3^ | 0.94 | 0.87 – 1.01 | 0.090 |
| Condition [Muscle Dystrophy]^3^ | 0.97 | 0.89 – 1.04 | 0.389 |
| Condition [Multiple Sclerosis]^3^ | 0.94 | 0.88 – 1.00 | 0.083 |
| Condition [Parkinson’s Disease]^3^ | 0.97 | 0.89 – 1.05 | 0.456 |
| Condition [Spinal Cord Injury]^3^ | 0.95 | 0.89 – 1.02 | 0.201 |
| Condition [Stroke]^3^ | 0.93 | 0.87 – 1.00 | 0.056 |
| Mobility Aid [Manual wheelchair]^4^ | 1.00 | 0.95 – 1.04 | 0.857 |
| Mobility Aid [Mobility scooter]^4^ | 0.98 | 0.92 – 1.04 | 0.428 |
| Mobility Aid [None]^4^ | 1.02 | 0.97 – 1.07 | 0.376 |
| Mobility Aid [Other]^4^ | 0.95 | 0.89 – 1.03 | 0.175 |
| Mobility Aid [Powered wheelchair]^4^ | 1.00 | 0.95 – 1.05 | 0.966 |
| Mobility Aid [Walking sticks]^4^ | 1.01 | 0.96 – 1.05 | 0.720 |
| Mobility Aid [Zimmer frame]^4^ | 1.00 | 0.95 – 1.06 | 0.914 |
| LTPA SCORE | 1.00 | 1.00 – 1.00 | 0.069 |
| Household activity SCORE | 1.00 | 1.00 – 1.00 | 0.693 |
| Work related activity SCORE | 1.00 | 1.00 – 1.01 | 0.461 |
| Sedentary Hours PerDay | 1.00 | 1.00 – 1.01 | 0.345 |
| Observations | 192 | | |
| R^2^ Nagelkerke | 0.203 | | |

*Abbreviations: LTPA = Leisure-time physical activity*

^1^*Reference: Female*

^2^*Reference: Government-issued isolation*

^3^*Reference: Cerebral Palsy*

^4^*Reference: Crutches*
