# Supplementary material for: Physical Activity and Health-Related Quality of Life in Adults With a Neurologically-Related Mobility Disability During the COVID-19 Pandemic: An Exploratory Analysis
Source: Front Neurol. 2021 Aug 27;12:699884. doi: 10.3389/fneur.2021.699884 (PMC8429606; doi:10.3389/fneur.2021.699884)
Supplement: Supplementary file 10 [file Table_9.docx]

| **Supplementary Table 9. Summary statistics of GLM: Vitality** | | | |
| --- | --- | --- | --- |
|  | **SVS SCORE** | | |
| *Predictors* | *Estimates* | *CI* | *p* |
| (Intercept) | 1.46 | 1.27 – 1.72 | **<0.001** |
| Age | 1.00 | 1.00 – 1.00 | 0.218 |
| Sex [male]^1^ | 0.99 | 0.94 – 1.05 | 0.775 |
| Sex [unknown]^1^ | 1.77 | 0.91 – 5.87 | 0.220 |
| Situation [none]^2^ | 1.07 | 0.93 – 1.26 | 0.371 |
| Situation [other]^2^ | 1.11 | 0.96 – 1.35 | 0.220 |
| Situation [self-imposed isolation]^2^ | 1.11 | 1.03 – 1.18 | **0.003** |
| Situation [social distancing]^2^ | 1.07 | 0.99 – 1.14 | 0.065 |
| Condition [Fibromyalgia]^3^ | 1.04 | 0.91 – 1.20 | 0.542 |
| Condition [Muscle Dystrophy]^3^ | 0.97 | 0.85 – 1.10 | 0.651 |
| Condition [Multiple Sclerosis]^3^ | 0.95 | 0.85 – 1.06 | 0.390 |
| Condition [Parkinson’s Disease]^3^ | 0.99 | 0.88 – 1.12 | 0.902 |
| Condition [Spinal Cord Injury]^3^ | 0.94 | 0.83 – 1.05 | 0.296 |
| Condition [Stroke]^3^ | 1.03 | 0.90 – 1.18 | 0.668 |
| Mobility Aid [Manual wheelchair]^4^ | 1.00 | 0.87 – 1.13 | 0.968 |
| Mobility Aid [Mobility scooter]^4^ | 1.25 | 0.99 – 1.62 | 0.075 |
| Mobility Aid [None]^4^ | 0.97 | 0.85 – 1.09 | 0.633 |
| Mobility Aid [Other]^4^ | 0.97 | 0.81 – 1.21 | 0.791 |
| Mobility Aid [Powered wheelchair]^4^ | 0.97 | 0.85 – 1.10 | 0.657 |
| Mobility Aid [Walking sticks]^4^ | 0.98 | 0.86 – 1.10 | 0.731 |
| Mobility Aid [Zimmer frame]^4^ | 1.02 | 0.88 – 1.19 | 0.782 |
| LTPA SCORE | 1.00 | 1.00 – 1.00 | 0.072 |
| Household activity SCORE | 1.00 | 1.00 – 1.00 | 0.502 |
| Work related activity SCORE | 1.00 | 0.99 – 1.01 | 0.543 |
| Sedentary Hours PerDay | 1.00 | 0.98 – 1.02 | 0.800 |
| Observations | 199 | | |
| R^2^ Nagelkerke | 0.184 | | |

*Abbreviations: LTPA = Leisure-time physical activity, SVS = Subjective*Vitality*Scales*

^1^*Reference: Female*

^2^*Reference: Government-issued isolation*

^3^*Reference: Cerebral Palsy*

^4^*Reference: Crutches*
