# Supplementary material for: Physical Activity and Health-Related Quality of Life in Adults With a Neurologically-Related Mobility Disability During the COVID-19 Pandemic: An Exploratory Analysis
Source: Front Neurol. 2021 Aug 27;12:699884. doi: 10.3389/fneur.2021.699884 (PMC8429606; doi:10.3389/fneur.2021.699884)
Supplement: Supplementary file 11 [file Data_Sheet_1.pdf]

School of Sport, Exercise & Rehabilitation Sciences

Impact of the COVID-19 Pandemic on Physical Activity and Health-Related Quality of Life in Adults with a Neurologically-Related Mobility Disability

Page 1 - Participant Information Sheet

Impact of Coronavirus Restrictions on Physical Activity and Wellbeing

You are invited to take part in this study exploring the effect of the coronavirus disease 2019 (COVID-19) pandemic on your physical activity behaviours. Whether or not you take part is your choice. If you do not want to take part, you do not have to give a reason. If you do want to take part now, but change your mind later, you can leave the study at any time by emailing the principal investigator.

Why are we doing the study?

COVID-19 can result in severe acute respiratory syndrome coronavirus 2 (SARS-CoV-2) and has widespread effects on the body. Governments around the world have imposed strict restrictions in order to reduce the spread of the disease and ‘flatten the curve’ to avoid overwhelming healthcare services. These restrictions include isolation and the practice of social distancing, which could unintentionally impact our exercise behaviours. Thus, the purpose of this study is to understand the effect of the COVID-19 pandemic on physical activity levels and health-related quality of life in adults with a neurologically-related mobility disability. We plan to capture changes over time as the UK Government alters their procedures to manage this pandemic. Information gathered will help us to better support individuals with physical disabilities, not only during future pandemics or serious global events, but during their everyday lives to engage in health promoting behaviours.

What is involved?

If you would like to take part in the study, we ask that you give your consent on page 2 and then complete a series of short online questionnaires about your physical activity patterns and health-related quality of life. These questionnaires should take about 25 minutes in total. Once you have completed the questionnaires, and providing you are happy to be involved in completing this survey in the future, we would like you to provide further consent for us to contact you at a later date. You will be invited to complete the survey on six occasions over the course of the next 48 weeks.

What are the benefits for taking part in the study?

There are no direct benefits to you from taking part in this study. However, this study may play an important role in understanding the effects of a global pandemic on health-behaviours in adults with a neurologically-related mobility disability. This information may not only help us better prepare for future events involving periods of increased isolation but manage the healthcare needs of individuals with ongoing neurological conditions and physical disabilities as society recovers over the coming months.

Are there any risks to being part of this study?

The risks involved in the study are low, although thinking about your physical activity behaviours and health-related quality of life might be a sensitive topic. Some people may find it stressful, particularly during this time.

If you are worried about any aspect of your physical or mental health then we advise you contact your doctor or relevant healthcare practitioner. In addition, below is contact information for UK organisations that provides emotional support for people experiencing distress.

The Samaritans (UK) – general mental health support, including suicidal and other distressing thoughts

https://www.samaritans.org/

MIND (UK) – mental health and COVID-19 mental health support

https://www.mind.org.uk/

What are the rights of participants in the study?

Taking part in this study is entirely voluntary. You are free to say no to participating, or to withdraw from the research study, at any time, without any disadvantage. You do not have to give a reason for your withdrawal. If you do withdraw from the study, we will retain the information about you that we have already obtained for analysis to achieve the objectives of this research study. It will not be used for any other purpose. Participation or withdrawal will not impact in any way the ongoing care you may be receiving now, remotely or in the future.

You have the right to access any information that we obtain on you from the study.

Results of the study?

Results may be published in an academic journal and/or presented at relevant conferences.

Confidentiality/anonymity and data security

All of your personal identifying information will be pseudonymised using a keycode. Any data presented in reports will be anonymous, that is information used in any journal article or presentation will not allow identification to any specific individual.

Data will be stored securely on a password protected computer to ensure confidentiality. Only the research team who are directly involved in the study will have access to the data.

We will follow ethical and legal practices in accordance with the Data Protection Act (2018), and the General Data Practice Regulations (GDPR) (2018).

Who is conducting this study?

Principal Investigator: Dr. Tom Nightingale, Lecturer in Exercise Physiology, School of Sport, Exercise and Rehabilitation Sciences, University of Birmingham. Email: T.E.Nightingale@bham.ac.uk

Co-investigators: Dr. Sally Fenton, Dr. Jet Veldhuijzen van Zaten & Dr. Nicola Heneghan, School of Sport, Exercise and Rehabilitation Sciences, University of Birmingham.

Ethical approval for this study has been given following review by the Science, Technology, Mathematics and Engineering (STEM) Ethical Review Committee at the University of Birmingham.

If you have any questions or concerns regarding the study please do not hesitate to contact: Dr. Tom Nightingale, Principal Investigator (T.E.Nightingale@bham.ac.uk).

If you are not satisfied with the response you receive, then please contact the STEM Research Ethics Manager: Susan Cottam (s.l.cottam@bham.ac.uk). ✱ *Required*

○ Continue to consent form/survey

Page 2 - Consent form

Before taking part in this study, I confirm that:

1. I have read and understood the Participant Information Sheet for the study ‘Impact of the COVID-19 Pandemic on Physical Activity and Health-Related Quality of Life in with a neurologically-related mobility disability

2. I have had sufficient time to consider whether or not to take part in the study and ask for advice if necessary

3. I am 18 years or older

4. I was born with or have sustained/developed a neurological condition (i.e. cerebral palsy, multiple sclerosis, Parkinson’s Disease, spinal cord injury, spina bifida or stroke etc.) resulting in upper and/or lower limb mobility impairments

4. I live in the United Kingdom (UK)

5. I understand that my participation is voluntary and that I am free to withdraw at any time, without giving any reason.

On this basis, I freely consent to participate in the above study ✱ *Required*

○ Yes

NB. Agreement is confirmed by clicking the 'next' button.

If you wish to withdraw from the study at any time, please send confirmation to T.E.Nightingale@bham.ac.uk.

General Demographics

How old are you (in years)? ✱ *Required*

Sex: ✱ *Required*

- Male
- Female
- Prefer not to say

What description best describes your current situation? ✱ *Required*

- Self-imposed isolation/shielded (considered at-risk)
- Isolation due to government legislation (i.e. working from home or furloughed)
- Practising social distancing
- None of the above
- Other

If you selected Other, please specify:

What is your ethnic group? Choose one option that best describes your ethnic background ✱ *Required*

- Caucasian/White
- Mixed/multiple ethnic groups
- Asian/Asian British
- Black/African/Caribbean/Black British
- Other

If you selected Other, please specify the ethnic group:

Do you have a clinical diagnosis which impacts your level of movement and physical function? ✱ *Required*

- Cerebral Palsy

- Multiple Sclerosis
- Parkinson's Disease
- Spinal Cord Injury
- Spina Bifida
- Stroke
- Other

If you selected Other, please specify the neurological condition:

How long have you had this clinical diagnosis/condition? *(please specify years and months, i.e. 4 years and 3 months)* ✱ *Required*

Please provide more information on your diagnosis, if known *(i.e. neurological level of spinal cord injury, complete or incomplete spinal cord injury, primary progressive or relapsing remitting Multiple Sclerosis, left or right sided paresthesia etc).*

What is the **main** *(more than 50% of the time)* mobility aid you use? ✱ *Required*

- Manual wheelchair
- Power wheelchair
- Mobility Scooter
- Zimmer frame
- Walking stick(s)
- Crutches
- None
- Other

If you selected Other, please specify:

Health Assessment Questionnaire (HAQ)

For each category, please check the **one** response that best describes your abilities over the **past 7 days**

Dressing and Grooming ✱ *Required*

	No Difficulty	Some Difficulty	Much Difficulty	Unable to do
Dress yourself, including tying shoelaces and doing buttons	☐	☐	☐	☐
Shampoo your hair	☐	☐	☐	☐

Rising ✱ *Required*

	No Difficulty	Some Difficulty	Much Difficulty	Unable to do
Stand up from an armless chair	☐	☐	☐	☐
Get in and out of bed	☐	☐	☐	☐

Eating ✱ *Required*

	No Difficulty	Some Difficulty	Much Difficulty	Unable to do
Cut your meat	☐	☐	☐	☐
Lift a full cup or glass to your mouth	☐	☐	☐	☐
Open a new carton of milk	☐	☐	☐	☐

Walking

Please don't select more than 1 answer(s) per row.

Please select at least 2 answer(s).

	No Difficulty	Some Difficulty	Much Difficulty	Unable to do
Walk outdoors on flat ground	☐	☐	☐	☐
Climb up five stairs	☐	☐	☐	☐

Hygiene

Please don't select more than 1 answer(s) per row.

Please select at least 3 answer(s).

	No Difficulty	Some Difficulty	Much Difficulty	Unable to do
Wash and dry your entire body	☐	☐	☐	☐
Take a bath	☐	☐	☐	☐
Get on and off the toilet	☐	☐	☐	☐

Reach ✱ *Required*

	No Difficulty	Some Difficulty	Much Difficulty	Unable to do
Reach and get down a 5 lb object (for example, a bag of sugar from just above your head).	☐	☐	☐	☐
Bend down to pick up clothing from the floor	☐	☐	☐	☐

Grip ✱ *Required*

	No Difficulty	Some Difficulty	Much Difficulty	Unable to do
Open car doors	☐	☐	☐	☐
Open Jars which have been previously opened	☐	☐	☐	☐
Turn taps on and off	☐	☐	☐	☐

Activities ✱ *Required*

	No Difficulty	Some Difficulty	Much Difficulty	Unable to do
Run errands and shop	☐	☐	☐	☐
Get in and out of a car	☐	☐	☐	☐
Do chores such as vacuuming, housework or light gardening	☐	☐	☐	☐

Do you **usually** *(more than 50% of the time)* use the following aids or devices for any of the activities listed above? *Check all that apply* *Optional*

- ☐ Canes
- ☐ Walker
- ☐ Crutches
- ☐ Wheelchair/scooter
- ☐ Raised toilet seat
- ☐ Bath seat
- ☐ Jar opener (for jars previously opened)
- ☐ Special or built-up utensils
- ☐ Special or built-up chair
- ☐ Bath rail
- ☐ Long-handled appliance for reach
- ☐ Other

If you selected Other, please specify:

Do you **usually** *(more than 50% of the time)* need help from another person for any of the following? *Check all that apply* *Optional*

- ☐ Errands and housework
- ☐ Reaching
- ☐ Dressing and grooming
- ☐ Gripping and opening things
- ☐ Eating
- ☐ Walking
- ☐ Rising
- ☐ Hygiene

Please check the number, from 0 to 10, which indicates how much **pain** you have had **over the past 7 days**, with 0 being *'no pain'* and 10 being *'pain as bad as it could be'*. ✱ *Required*

	0	1	2	3	4	5	6	7	8	9	10
Pain Scale Rating	☐	☐	☐	☐	☐	☐	☐	☐	☐	☐	☐

Physical Activity Scale for Individuals with Physical Disabilities (PASIPD)

Instructions: This questionnaire is about your current level of physical activity and exercise. Please remember there are no right or wrong answers. We simply need to assess your current level of activity.

Leisure Time Activity

1. During the **past 7 days** how often did you engage in **stationary activities** such as reading, watching TV, computer games, or doing arts and crafts? ✱ *Required*

- Never (Go to question #2)
- Seldom (1-2d)
- Sometimes (3-4d)
- Often (5-7d)

What were these activities? *Optional*

On average, how many ***hours per day*** did you spend in these ***stationary activities*?**

- Less than 1 hr
- 1 but less than 2 hr
- 2 - 4 hr
- More than 4 hr

2. During the ***past 7 days***, how often did you ***walk, wheel, push outside*** your home ***other than specifically for exercise***. For example, getting to work or class, taking the dog out, shopping, or other errands? ✱ *Required*

- Never (Go to question #3)
- Seldom (1-2d)
- Sometimes (3-4d)
- Often (5-7d)

On average, how many ***hours per day*** did you spend ***walking, wheeling or pushing outside*** your home?

- Less than 1 hr
- 1 but less than 2 hr

- 2 - 4 hr
- More than 4 hr

3. During the ***past 7 days***, how often did you engage in ***light sport or recreational activities*** such as bowling, golf with a cart, hunting or fishing, darts, billiards or pool, therapeutic exercise (physical or occupational therapy, stretching, use of a standing frame) or other similar activities? ✱ *Required*

- Never (Go to question #4)
- Seldom (1-2d)
- Sometimes (3-4d)
- Often (5-7d)

What were these activities?

On average, how many ***hours per day*** did you spend in these ***light sport or recreational*** **activities?**

- Less than 1 hr
- 1 but less than 2 hr
- 2 - 4 hr
- More than 4 hr

4. During the ***past 7 days***, how often did you engage in ***moderate sport and recreational*** activities such as doubles tennis, softball, golf without a cart, ballroom dancing, wheeling or pushing for pleasure or other similar activities? ✱ *Required*

- Never (Go to question #5)
- Seldom (1-2d)
- Sometimes (3-4d)
- Often (5-7d)

What were these activities?

On average, how many ***hours per day*** did you spend in these ***moderate sport and recreational*** activities?

- Less than 1 hr
- 1 but less than 2 hr
- 2 - 4 hr
- More than 4 hr

5. During the ***past 7 days***, how often did you engage in ***strenuous sport and recreational*** activities such as jogging, wheelchair racing (training), off-road pushing, swimming, aerobic dance, arm cranking, cycling (hand or leg), singles tennis, rugby, basketball, walking with crutches and braces, or other similar activities ✱ *Required*

- Never (Go to question #6)
- Seldom (1-2d)
- Sometimes (3-4d)
- Often (5-7d)

What were these activities?

On average, how many ***hours per day*** did you spend in these ***strenuous sport and recreational*** activities?

- Less than 1 hr
- 1 but less than 2 hr
- 2 - 4 hr
- More than 4 hr

6. During the ***past 7 days***, how often did you do any exercise ***specifically to increase muscle strength and endurance*** such as lifting weights, push-ups, pull-ups, dips, or wheel- chair push-ups, etc? ✱ *Required*

- Never (Go to question #7)
- Seldom (1-2d)
- Sometimes (3-4d)
- Often (5-7d)

What were these activities?

On average, how many ***hours per day*** did you spend in these ***exercises to increase muscle strength and endurance***?

- Less than 1 hr
- 1 but less than 2 hr
- 2 - 4 hr
- More than 4 hr

Household Activity

7. During the ***past 7 days***, how often have you done any ***light housework***, such as dusting, sweeping floors or washing dishes? ✱ *Required*

- Never (Go to question #8)
- Seldom (1-2d)
- Sometimes (3-4d)
- Often (5-7d)

On average, how many ***hours per day*** did you spend doing ***light housework***?

- Less than 1 hr
- 1 but less than 2 hr
- 2 - 4 hr
- More than 4 hr

8. During the ***past 7 days***, how often have you done any ***heavy housework or chores*** such as vacuuming, scrubbing floors, washing windows or walls etc? ✱ *Required*

- Never (Go to question #9)
- Seldom (1-2d)
- Sometimes (3-4d)
- Often (5-7d)

On average, how many ***hours per day*** did you spend doing ***heavy housework or chores***?

- Less than 1 hr

- 1 but less than 2 hr
- 2 - 4 hr
- More than 4 hr

9. During the ***past 7 days***, how often have you done ***home repairs*** like carpentry, painting, furniture refinishing, electrical work, etc? ✱ *Required*

- Never (Go to question #10)
- Seldom (1-2d)
- Sometimes (3-4d)
- Often (5-7d)

On average, how many ***hours per day*** did you spend doing ***home repairs***?

- Less than 1 hr
- 1 but less than 2 hr
- 2 - 4 hr
- More than 4 hr

10. During the ***past 7 days*** how often have you done ***lawn work or yard care*** including mowing, leaf or snow removal, tree or bush trimming, or wood chopping, etc? ✱ *Required*

- Never (Go to question #11)
- Seldom (1-2d)
- Sometimes (3-4d)
- Often (5-7d)

On average, how many ***hours per day*** did you spend doing ***lawn work or yard care***?

- Less than 1 hr
- 1 but less than 2 hr
- 2 - 4 hr
- More than 4 hr

11. During the ***past 7 days***, how often have you done ***outdoor gardening***? ✱ *Required*

- Never (Go to question #12)
- Seldom (1-2d)
- Sometimes (3-4d)

- Often (5-7d)

On average, how many ***hours per day*** did you spend doing ***outdoor gardening***?

- Less than 1 hr
- 1 but less than 2 hr
- 2 - 4 hr
- More than 4 hr

12. During the ***past 7 days***, how often did you ***care for another person***, such as children, a dependent spouse, or another adult? ✱ *Required*

- Never (Go to question #13)
- Seldom (1-2d)
- Sometimes (3-4d)
- Often (5-7d)

On average, how many ***hours per day*** did you spend ***caring for another person***?

- Less than 1 hr
- 1 but less than 2 hr
- 2 - 4 hr
- More than 4 hr

Work-Related Activity

13. During the ***past 7 days***, how often were ***physically active*** while ***working for for pay or as a volunteer***? (Exclude work that mainly involved sitting with slight arm movement such as light office work, computer work, light assembly line work, driving bus or van, etc.) ✱ *Required*

- Never
- Seldom (1-2d)
- Sometimes (3-4d)
- Often (5-7d)

On average, how many ***hours per day*** were you active while ***working for pay or as a volunteer***?

- Less than 1 hr
- 1 but less than 2 hr
- 2 - 4 hr

- More than 4 hr

Are you currently ***leaving your house to go to go work***? ✱ *Required*

- Never
- Seldom (1-2d)
- Sometimes (3-4d)
- Often (5-7d)

On average, how many ***hours per day*** did you spend ***out of your house at work***?

- Less than 1 hr
- 1 but less than 2 hr
- 2 - 4 hr
- More than 4 hr

Additional Physical Activity Related Questions

How have your physical activity levels ***changed*** compared to before the COVID-19 pandemic?

- Considerably less
- Slightly less
- About the same
- Slightly more
- Considerably more

Do you use a wearable fitness tracker *e.g. Fitbit*?

- Yes
- No

If yes, please specify the make/model of your wearable *(i.e. Apple Watch, Series 5 or Fitbit Charge 4 etc.)*:

If you have been wearing this device, please provide a rough breakdown of your physical activity levels over the previous 7 days. These can be any metrics that are most relevant to you but may include: *minutes of moderate-to-vigorous physical activity per week, energy expenditure, sedentary time, activity minutes, calorie burn etc.*

Fear of COVID-19

The below questions relate to your feelings around COVID-19. *Please select one option for each statement.* ✱ *Required*

	Strongly disagree	Disagree	Neither agree nor disagree	Agree	Strongly Agree
1. I am most afraid of coronavirus-19	☐	☐	☐	☐	☐
2. It makes me uncomfortable to think about coronavirus-19	☐	☐	☐	☐	☐
3. My hands become clammy when I think about coronavirus-19	☐	☐	☐	☐	☐
4. I am afraid of losing my life because of coronavirus-19	☐	☐	☐	☐	☐
5. When watching news and stories about coronavirus-19 on social media, I become nervous or anxious	☐	☐	☐	☐	☐
6. I cannot sleep because I'm worrying about getting coronavirus-19	☐	☐	☐	☐	☐
7. My heart races or palpitates when I think about getting coronavirus-19	☐	☐	☐	☐	☐

UCLA Loneliness Scale

Instructions: The following statements describe how people sometimes feel. For each statement, please indicate how often you feel the way described over the ***past 7 days*** by selecting the appropriate response. Here is an example: How often do you feel happy? If you never felt happy, you would respond "never", if you always feel happy, you would respond "always". ✱ *Required*

	Never	Rarely	Sometimes	Always
1. How often do you feel that you are "in tune" with the people around you?	☐	☐	☐	☐
2. How often do you feel that you lack companionship?	☐	☐	☐	☐
3. How often do you feel that there is no one you can turn to?	☐	☐	☐	☐
4. How often do you feel alone?	☐	☐	☐	☐
5. How often do you feel part of a group of friends?	☐	☐	☐	☐
6. How often do you feel that you have a lot in common with the people around you?	☐	☐	☐	☐
7. How often do you feel that you are no longer close to anyone?	☐	☐	☐	☐
8. How often do you feel that your interests and ideas are not shared by those around you?	☐	☐	☐	☐
9. How often do you feel outgoing and friendly?	☐	☐	☐	☐
10. How often do you feel close to people?	☐	☐	☐	☐
11. How often do you feel left out?	☐	☐	☐	☐
12. How often do you feel that your relationships with others are not meaningful?	☐	☐	☐	☐
13. How often do you feel that no one really knows you well?	☐	☐	☐	☐
14. How often do you feel isolated from others?	☐	☐	☐	☐
15. How often do you feel you can find companionship when you want it?	☐	☐	☐	☐
16. How often do you feel that there are people who really understand you?	☐	☐	☐	☐
17. How often do you feel shy?	☐	☐	☐	☐
18. How often do you feel that people are around you but not with you?	☐	☐	☐	☐
19. How often do you feel that there are people you can talk to?	☐	☐	☐	☐
20. How often do you feel that there are people you can turn to?	☐	☐	☐	☐

Subjective Vitality Scale

Below are 6 statements that relate to how you feel in your day-to-day life. Please respond to each of these statements by indicating the degree to which the statement is true in terms of how you have been feeling over the ***past 7 days***. ✱ *Required*

	1. Not at all true	2.	3.	4. Somewhat true	5.	6.	7. Very true
1. I have been feeling alive and vital	☐	☐	☐	☐	☐	☐	☐
2. I have been feeling so alive I just want to burst	☐	☐	☐	☐	☐	☐	☐
3. I have had energy and spirit	☐	☐	☐	☐	☐	☐	☐
4. I have looked forward to each new day	☐	☐	☐	☐	☐	☐	☐
5. I nearly always felt alert and awake	☐	☐	☐	☐	☐	☐	☐
6. I have been feeling energized	☐	☐	☐	☐	☐	☐	☐

Fatigue Severity Scale (FSS)

Please check the number between 1 and 7 which you feel best fits the following statements. This refers to your usual way of life ***within the last 7 days***. 1 indicates "strongly disagree" and 7 indicates "strongly agree". ✱ *Required*

	1. Strongly disagree	2.	3.	4.	5.	6.	7. Strongly agree
1. My motivation is lower when I am fatigued	☐	☐	☐	☐	☐	☐	☐
2. Exercise brings on my fatigue	☐	☐	☐	☐	☐	☐	☐
3. I am easily fatigued	☐	☐	☐	☐	☐	☐	☐
4. Fatigue interferes with my physical functioning	☐	☐	☐	☐	☐	☐	☐
5. Fatigue causes frequent problems for me	☐	☐	☐	☐	☐	☐	☐
6. My fatigue prevents sustained physical functioning	☐	☐	☐	☐	☐	☐	☐
7. Fatigue interferes with carrying out certain duties and responsibilities	☐	☐	☐	☐	☐	☐	☐
8. Fatigue is among my most disabling symptoms	☐	☐	☐	☐	☐	☐	☐
9. Fatigue interferes with my work, family or social life	☐	☐	☐	☐	☐	☐	☐

Visual Analogue Fatigue Scale

Please mark on the number line which describes your ***global fatigue*** with 0 being worst and 10 being normal within the ***last 7 days***. ✱ *Required*

	0	1	2	3	4	5	6	7	8	9	10
Global Fatigue	☐	☐	☐	☐	☐	☐	☐	☐	☐	☐	☐

Hospital Anxiety and Depression Scale (HADS)

Tick the box beside the reply that is closest to how you have been feeling in the ***last 7 days***. Don't take too long over your replies: your immediate is best.

I feel tense or 'wound up': ✱ *Required*

- Most of the time
- A lot of the time
- From time to time, occasionally
- Not at all

I still enjoy the things I used to enjoy: ✱ *Required*

- Definitely as much
- Not quite so much
- Only a little
- Hardly at all

I get a sort of frightened feeling as if something awful is about to happen: ✱ *Required*

- Very definitely and quite badly
- Yes, but not too badly
- A little, but it doesn't worry me
- Not at all

I can laugh and see the funny side of things: ✱ *Required*

- As much as I always could
- Not quite so much now
- Definitely not so much now
- Not at all

Worrying thoughts go through my mind: ✱ *Required*

- A great deal of the time
- A lot of the time
- From time to time, but not too often
- Only occasionally

I feel cheerful: ✱ *Required*

- Not at all
- Not often
- Sometimes
- Most of the time

I can sit at ease and feel relaxed: ✱ *Required*

- Definitely
- Usually
- Not often
- Not at all

I feel as if I am slowed down: ✱ *Required*

- Nearly all the time
- Very often
- Sometimes
- Not at all

I get a sort of frightened feeling like 'butterflies' in the stomach: ✱ *Required*

- Not at all
- Occasionally
- Quite often
- Very often

I have lost interest in my appearance: ✱ *Required*

- Definitely
- I don't take as much care as I should
- I may not take quite as much care
- I take just as much care as ever

I feel restless as I have to be on the move: ✱ *Required*

- ○ Very much indeed
- ○ Quite a lot
- ○ Not very much
- ○ Not at all

I look forward with enjoyment to things: ✱ *Required*

- ○ As much as I ever did
- ○ Rather less than I used to
- ○ Definitely less than I used to
- ○ Hardly at all

I get sudden feelings of panic: ✱ *Required*

- ○ Very often indeed
- ○ Quite often
- ○ Not very often
- ○ Not at all

I can enjoy a good book or radio or TV program: ✱ *Required*

- ○ Often
- ○ Sometimes
- ○ Not often
- ○ Very seldom

If you found any of the above questions unsettling or challenging to complete, particularly during this difficult time, and are worried about your mental health, then we would like to remind you of the various UK organisations that can help provide emotional support:

The Samaritans (UK) – general mental health support, including suicidal and other distressing thoughts

https://www.samaritans.org/

MIND (UK) – mental health and COVID-19 mental health support

https://www.mind.org.uk/

Additional Questions

There are no right or wrong answers to these questions. Please complete with what seems most relevant to you at this specific moment in time.

1. What have you found most challenging during the COVID-19 pandemic?

2. What have you found to be the ***biggest barrier or obstacle to perform exercise or physical activity at this time***?

3. What have you found to be the ***biggest facilitator (or motivator) to perform exercise or physical activity at this time***?

Follow-up

Would you be willing to complete this questionnaire pack again in 8 weeks' time so we can capture changes in these outcomes?

- Yes
- No

If yes, please provide your email address so that we can send you a reminder.

Please re-type your above email address

Final page

We thank you for your time spent taking this survey.

Your response has been recorded.
