# Supplementary material for: Physical Activity and Health-Related Quality of Life in Adults With a Neurologically-Related Mobility Disability During the COVID-19 Pandemic: An Exploratory Analysis
Source: Front Neurol. 2021 Aug 27;12:699884. doi: 10.3389/fneur.2021.699884 (PMC8429606; doi:10.3389/fneur.2021.699884)
Supplement: Supplementary file 13 [file Data_Sheet_3.docx]

**APPENDIX C – Supplementary materials – METHODS & RESULTS**

**METHODS**

**Validated tools used to capture Patient Reported Outcome Measures (PROMs)**

***Functional disability and pain***

The degree of functional disability was determined via the Health Assessment Questionnaire (HAQ), which comprises 8 sections (with two or three questions in each section) that reflect activities of daily living (i.e., dressing, rising, eating, walking, hygiene, grip, reach, and activities) [1]. Participants were asked to rate their ability over the past 7-days to perform specific activities (e.g., “shampoo your hair”, “climb up five stairs”, *the full list of 20 questions can be found in Appendix A*) on a scale ranging from “no difficulty” (scored as 0) to “unable to do” (scored as 3). Participants also indicated if they usually used aids/devices (e.g., “wheelchair/scooter”, “raised toilet seat”) or required assistance from another person to perform the aforementioned activities of daily living. For each section, the overall score given is the worst score reported within that section, i.e., if one question is scored 1 and another 2, then the score for the section is 2. Furthermore, if an aid/device is used or if help is required from another individual, then the minimum score for that section is computed as 2 (unless the score is already 3, i.e., scores of 0 or 1 are increased to 2). The standard disability index (SDI) is calculated as the sum of the computed category scores, divided by the number of categories answered. Consequently, this score ranges from 0 to 3, with a higher score representing higher functional disability (i.e., 0 = no disability and 3 = total dependence on others). HAQ-SDI scores >1 are generally considered to indicate the presence of disability. The HAQ is considered a generic rather than a disease-specific instrument, which lends itself to be used across our diverse sample. In addition, the HAQ-SDI has also demonstrated acceptable convergent validity when compared to other clinical and laboratory measures [2].

Pain was measured using an 11-point numerical rating scale (ranging from, 0 = “no pain” to 10 = “pain as bad as it could be”). Numerical rating scales to measure pain have been found to be reliable and demonstrate good face and criterion validity, and they are recommended as a core outcome measure for chronic pain clinical trials by the Initiative on Methods, Measurement, and Pain Assessment in Clinical Trials (IMMPACT) [3; 4].

***Physical activity and sedentary behaviour***

Self-reported physical activity was determined using the physical activity scale for individuals with physical disabilities (PASIPD) [5]. This 13-item questionnaire was adapted from the Physical Activity Scale for the Elderly and follows a similar format to that of the International Physical Activity Questionnaire. Briefly, the PASIPD asks participants to report the number of days per week and hours per day of engagement in leisure-time (6 items), household (6 items) and work-related (1-item) activities over the preceding 7 days. These specific questions can be found in the online survey (Appendix A). The average hours per day spent performing each item was multiplied by a metabolic equivalent (MET) value associated with the intensity of each activity [5]. These values were summed over items within each category and in total to derive composite scores, expressed as MET hr/d. The test-retest reliability and criterion validity of the PASIPD has been shown to be comparable to that of well-established self-report physical activity questionnaires commonly used in the general population [6]. Participants were also asked how their physical activity levels have changed compared to before the COVID-19 pandemic. Changes in physical activity levels were captured using a five-point Likert scale: “*slightly more*”, “*considerably more*”, “*about the same*”, “*slightly less*”, “*considerably less*”.

**Fear of COVID-19**

Total fear of COVID-19 score was determined via the reliable and validated 7-item Fear of COVID-19 Scale [7]. Participants indicated their level of agreement with statements (i.e., I cannot sleep because I’m worrying about getting coronavirus-19) according to a 5-item Likert scale: “strongly disagree,” “disagree,” “neither agree nor disagree,” “agree,” and “strongly agree”. These items were scored from 1 to 5 and summed together to generate a total score, ranging from 7 (low fear) to 35 (greatest fear).

**Loneliness**

Loneliness was determined via the 20-item UCLA Loneliness Scale (Version 3) [8]. Participants were asked to rate (1 = “never”, 2 = “rarely”, 3 = “sometimes”, 4 = “always”) how often they feel the way described (i.e., how often do you feel left out?). Nine items were reverse scored and the scores for each item then summed together. Higher scores indicate greater levels of loneliness. The UCLA Loneliness Scale (Version 3) is highly reliable and demonstrates acceptable convergent and construct validity [8]. This scale has previously been used to assess loneliness in older people with cerebral palsy [9] and individuals with spinal cord injury [10].

**Subjective vitality**

Vitality experienced during the previous 7 days was measured using the 6-item Subjective Vitality Scale (SVS) [11]. Participants were asked to rate statements (e.g., “I have been feeling alive and vital”) on a scale from 1 (“not at all true”) to 7 (“very true”). Subjective vitality is an indicator of eudaimonic wellbeing and is conceptualized as a specific and subjective positive psychological state (defined as a sense of feeling alive, vital, and full of energy) [11]. Total scores are averaged across the 7 items, ranging from 1 – 7, with higher scores indicating better eudaimonic wellbeing.

**Fatigue**

The 9-item Fatigue Severity Scale (FSS) was administered to measure the severity of fatigue and its effects on certain behaviours [12]. Each item is scored on a 7-point Likert scale, “1 = strongly disagree” and “7 = strongly agree”. Composite scores range from 9 to 63, with higher scores indicating a greater fatigue severity. A global fatigue visual analogue scale was also administered, with 0 being worst imaginable and 10 being normal. The FSS is the most frequently used measurement tool to assess fatigue, and its validity and reliability has been demonstrated in individuals with neurological conditions (stroke [13] and spinal cord injury [14]).

**Anxious and depressive symptoms**

The Hospital Anxiety and Depression Scale (HADS) was used to determine the prevalence of anxious (7 items) and depressive (7 items) symptoms [15]. Participants were asked how they felt during the previous 7 days, indicating their agreement (on a scale from 0 to 3) with specific statements (i.e., “I get sudden feelings of panic” or “I feel cheerful”). The HADS has previously demonstrated good validity in individuals with neurological conditions (Parkinson’s disease [16] and multiple sclerosis [17]). Scores across the respective 7 items for anxious and depressive symptoms were summed together and the following scoring thresholds utilised: 0 – 7 = Normal, 8 – 10 = Borderline abnormal (borderline case), 11 – 21 = Abnormal (case).

**Additional E-survey Information**

The below information satisfies components of the checklist for Reporting Results of Internet E-Surveys (CHERRIES) [18] that were not included in the manuscript due to page limit restrictions. The open e-survey was voluntary and there were no incentives offered. Items were not randomized or alternated in the e-survey and were delivered in the order shown in Appendix A. Where possible, adaptive questioning was used to reduce the number and complexity of the questions (e.g., for the PASIPD). The below template was used to advertise the survey on social media via various national charities and organisations (examples provided in the acknowledgements section):

*‘Individuals living in the UK with a neurologically-related mobility disability. Please take a look at this short survey to understand changes in your physical activity and wellbeing as a result of COVID-19 (link). RT’s welcome (@charities/organisation)’.*

The e-survey was distributed across 14 pages (including participant information sheet, consent and thank you page), with a mean of 13 items (range: 3 - 33) per page. Respondents were able to change their answers using a back button if necessary. Online surveys (formerly BOS) is designed to protect respondent anonymity and this platform does not use cookies to assign a unique user identifier to each computer accessing the survey. Additionally, it was not possible to access any information about respondents’ IP addresses. Consequently, we were unable to identify unique site visitors, which precluded us from calculating the view rate. The only strategy implemented to identify multiple entries was a rudimentary log file analysis, where data was visually assessed to identify duplicate entries based on identical generic demographic information (e.g., age, sex, condition, ethnic group, length of diagnosis). Despite not being able to count unique visitors to the e-survey, the participation rate (ratio of visitors who agreed to participate/unique first survey page visitors) was calculated as 9.4%. Furthermore, the completion rate (ratio of users who finished the survey/users who agreed to participate) was 59.8%. Respondent progress through the e-survey is described below in Figure 1.


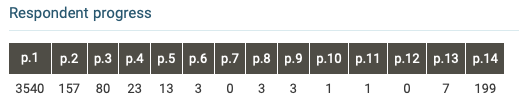


**Figure 1. Respondents progress through the e-survey.** The first page was viewed 3,540 times. 333 individuals provided informed consent and 199 individuals completed the e-survey.

**RESULTS**

**Expanded free text data from open ended questions**

The following table offers some quotes taken from the open-ended free text questions. These are illustrative of the main categories (>20 participants) that emerged from the content analysis.

**Table 1: Biggest challenges encountered during the COVID-19 pandemic**

| **Lack of normal life/lockdown (n=91)** | *‘Having to stay indoors most of the time’ (P29)*  *‘Not being able to do what I would usually do from day to day’ (P08)*  *‘I had a carefully built up a programme of exercise classes, social contact, political meetings, further education classes, arts and theatre visits to keep me fit and mentally active. That all stopped abruptly. I have tried to find alternatives but it has been difficult’ (P48)*  *‘No end date in sight when it will be safe to re-enter society and get back to a new normal’ (P05)* |
| --- | --- |
| **Missing or not seeing family (n=44)** | *‘Not seeing family frequently enough’ (P167)*  *‘Being separated from family members’ (P125)*  *‘Not being able to physically meet family & friends’ (P52)*  *‘Not seeing grandchildren.’ (P66)* |
| **‘Fear/uncertainty’ or ‘isolation’ (n=30)** | *‘Tragic loss of life, lack of govt clarity & people's disinterest in social distancing is very upsetting’ (P51)*  *‘The isolation and lack of people contact.’ (P18)*  *‘Isolation and fear of getting the virus’ (P32)*  *‘Feeling scared to go outside’ (P130)* |
| **Other issues related to ‘food/bills’ (n=27)** | *‘Getting online shopping slots’ (P157)*  *‘Sourcing food and paying bills’ (P07)*  *‘Trying to get support for shopping and other household duties’ (P44)*  *‘Purchasing goods/materials’ (P50)* |
| **Impact on exercise & leisure physical activities (n=21)** | *‘Lack of opportunity to sail (Sailability)’ (P194)*  *‘Lockdown and not going to the gym and attending my sports club’ (P54)*  *‘Not being able to go to the stables to do the horse’ (P61)*  *‘Lack of access to exercise (my gym instructor & equipment) and generally walking when at work, lack of care from Neurologist and MS Nurse team’ (P26)* |

**Table 2: Biggest barriers or obstacle to perform exercise or physical activity during the COVID-19 pandemic**

| **Closed gyms/pool/organised sport (n=53)** | *‘Accessible facilities, all being closed and no sign of re-opening’(P54)*  *‘Unable to visit my yoga group’ (P99)*  *‘My local leisure centre has been closed. I participate in 5 instructor led sessions per week.’ (P106)*  *‘Used to attend gym, classes etc regularly; these are not now available’ (P119)* |
| --- | --- |
| **Lack of motivation (n=30)** | *‘I’m not a good self-motivator for exercise at home’ (P116)*  *‘I find it better to do exercise with other people, not on my own at home’ (P139)*  *‘No motivation and training being cancelled.’ (P78)*  *‘Motivating myself’ (P95)* |
| **Fatigue (n=25)** | *‘Tired and fatigue with pain’ (P42)*  *‘Fatigue and time from caring duties /looking after poorly spouse’ (P52)*  *‘My conditions have been particularly bad due to changes in meds brought on by the pandemic. It has increased my fatigue. I'm barely able to get up out of bed and prepare and eat food once a day’. (P62)*  *‘lack of mobility and fatigue’ (P192)* |
| **Leaving home (n=24)** | *‘Lack of open places to go’ (P63)*  *‘I live in a very small flat with a small garden. I've supposed to stay at home until now, so no going out!’ (P79)*  *‘Stuck in house and rear garden - limited mobility options and cannot get to outdoor spaces to lift spirit OR to physiotherapy sessions’ (P111)*  *‘Not being able to leave the house for exercise during earlier lockdown as needed level area near parking and non essential travel forbidden. Didn't feel I could even leave the house in wheelchair pushed by carer as feared being judged or abused as not technically exercising. Stress of pandemic and restricted to staying home exhausted me so home exercise became very difficult’. (P68)* |
| **Lack of equipment/space/support (n=24)** | *‘Finding somewhere to walk which is not crowded’ (P84)*  *‘Difficulty arranging equipment for exercise within my home’ (P149)*  *‘Not being able to get outside unaided’ (P195)*  *‘Space and equipment’ (P12)* |
| **Fear/including hurting/pain (n=22)** | *‘The pain resulting from exercise’ (P21)*  *‘Concern of falling and ending up in hospital.’ (P133)*  *‘Pools closed and walking outside is frightening as I had a rotator cuff injury for a couple of years following a fall. I fall more easily these days and the thought makes me anxious.’ (P175)*  *‘Exercise is very difficult for me but higher pain levels made it worse than normal’. (P77)* |

**Table 3: Biggest facilitator (or motivator) to perform exercise or physical activity during the COVID-19 pandemic**

| **Health (mental and physical)/weight (n=60)** | *‘Being made sedentary and the fear of obesity ‘ (P21)*  *‘The fact that the more I exercise, the easier it is to keep my limbs moving usefully to me.’ (P143)*  *‘It makes me feel so much better when I have taken part in a physical activity.’ (P145)*  *‘Trying to keep fit and healthy’ (P161)* |
| --- | --- |
| **Family/Healthcare practitioner support (n=30)** | *‘If my children are interested, I love getting exercise with them and feel there is a twofold reason to go’ (P47)*  *‘Partner, Pilates teacher and own determination’ (P39)*  *‘My physio ringing me at home’ (P66)*  *‘Phone calls for diabetic nurse to check up on me.’ (P150)* |
| **Online classes (n=28)** | *‘Online exercise classes with therapists’ (P108)*  *‘Virtual classes online (hurrah for Zoom!)’ (P106)*  *‘Really good regular online seated exercise class’ (P65)*  *‘An online class that was offered during lockdown on social media. Early on this helped’ (P170)* |
| **Leaving the house/fresh air/garden’ (n=27)** | *‘If the weather is nice and my daughter or husband is available then I want to go a little walk’ (P182)*  *‘When its sunny I feel motivated to be in the garden and walk about a little with my rollator’ (P127)*  *‘Making myself walk round the back garden’ (P58)*  *‘Getting out into nature and being outside’ (P37)* |

**RESOURCES**

**Inclusive exercise options**

•WheelPower online workouts (UK):<https://www.wheelpower.org.uk/sport-events>

•SCOPE = Equality for Dsiabled People, Exercising from home (UK): <https://www.scope.org.uk/advice-and-support/exercising-during-coronavirus/>

•NCHPAD Building Healthy Inclusive Communities [(USA): https://www.nchpad.org/Articles/9/Exercise~and~Fitness](https://www.nchpad.org/Articles/9/Exercise~and~Fitness)

•FREE Virtual Adaptive & Functional Fitness Training classes for Tetraplegics and Paraplegics [(USA): https://norcalsci.org/news/2020/10/18/adaptive-fitness-class](https://norcalsci.org/news/2020/10/18/adaptive-fitness-class)

**APPENDIX C - REFERENCES**

[1] J.F. Fries, P.W. Spitz, and D.Y. Young, The dimensions of health outcomes: the health assessment questionnaire, disability and pain scales. J Rheumatol 9 (1982) 789-93.

[2] B. Bruce, and J.F. Fries, The Stanford Health Assessment Questionnaire: dimensions and practical applications. Health Qual Life Outcomes 1 (2003) 20.

[3] D.C. Turk, R.H. Dworkin, D. Revicki, G. Harding, L.B. Burke, D. Cella, C.S. Cleeland, P. Cowan, J.T. Farrar, S. Hertz, M.B. Max, and B.A. Rappaport, Identifying important outcome domains for chronic pain clinical trials: an IMMPACT survey of people with pain. Pain 137 (2008) 276-285.

[4] R.H. Dworkin, D.C. Turk, J.T. Farrar, J.A. Haythornthwaite, M.P. Jensen, N.P. Katz, R.D. Kerns, G. Stucki, R.R. Allen, N. Bellamy, D.B. Carr, J. Chandler, P. Cowan, R. Dionne, B.S. Galer, S. Hertz, A.R. Jadad, L.D. Kramer, D.C. Manning, S. Martin, C.G. McCormick, M.P. McDermott, P. McGrath, S. Quessy, B.A. Rappaport, W. Robbins, J.P. Robinson, M. Rothman, M.A. Royal, L. Simon, J.W. Stauffer, W. Stein, J. Tollett, J. Wernicke, J. Witter, and Immpact, Core outcome measures for chronic pain clinical trials: IMMPACT recommendations. Pain 113 (2005) 9-19.

[5] R.A. Washburn, W. Zhu, E. McAuley, M. Frogley, and S.F. Figoni, The physical activity scale for individuals with physical disabilities: development and evaluation. Arch Phys Med Rehabil 83 (2002) 193-200.

[6] H.P. van der Ploeg, K.R. Streppel, A.J. van der Beek, L.H. van der Woude, M. Vollenbroek-Hutten, and W. van Mechelen, The Physical Activity Scale for Individuals with Physical Disabilities: test-retest reliability and comparison with an accelerometer. J Phys Act Health 4 (2007) 96-100.

[7] D.K. Ahorsu, C.Y. Lin, V. Imani, M. Saffari, M.D. Griffiths, and A.H. Pakpour, The Fear of COVID-19 Scale: Development and Initial Validation. Int J Ment Health Addict (2020) 1-9.

[8] D.W. Russell, UCLA Loneliness Scale (Version 3): reliability, validity, and factor structure. J Pers Assess 66 (1996) 20-40.

[9] S. Balandin, N. Berg, and A. Waller, Assessing the loneliness of older people with cerebral palsy. Disabil Rehabil 28 (2006) 469-79.

[10] N. Santino, V. Larocca, S.L. Hitzig, S.J.T. Guilcher, B.C. Craven, and R.L. Bassett-Gunter, Physical activity and life satisfaction among individuals with spinal cord injury: Exploring loneliness as a possible mediator. J Spinal Cord Med (2020) 1-7.

[11] R.M. Ryan, and C. Frederick, On energy, personality, and health: subjective vitality as a dynamic reflection of well-being. J Pers 65 (1997) 529-65.

[12] L.B. Krupp, N.G. LaRocca, J. Muir-Nash, and A.D. Steinberg, The fatigue severity scale. Application to patients with multiple sclerosis and systemic lupus erythematosus. Arch Neurol 46 (1989) 1121-3.

[13] O. Ozyemisci-Taskiran, E.B. Batur, S. Yuksel, M. Cengiz, and G.K. Karatas, Validity and reliability of fatigue severity scale in stroke. Top Stroke Rehabil 26 (2019) 122-127.

[14] H.A. Anton, W.C. Miller, and A.F. Townson, Measuring fatigue in persons with spinal cord injury. Arch Phys Med Rehabil 89 (2008) 538-42.

[15] A.S. Zigmond, and R.P. Snaith, The hospital anxiety and depression scale. Acta Psychiatr Scand 67 (1983) 361-70.

[16] F. Mondolo, M. Jahanshahi, A. Grana, E. Biasutti, E. Cacciatori, and P. Di Benedetto, The validity of the hospital anxiety and depression scale and the geriatric depression scale in Parkinson's disease. Behav Neurol 17 (2006) 109-15.

[17] R.A. Marrie, L. Zhang, L.M. Lix, L.A. Graff, J.R. Walker, J.D. Fisk, S.B. Patten, C.A. Hitchon, J.M. Bolton, J. Sareen, R. El-Gabalawy, J.J. Marriott, and C.N. Bernstein, The validity and reliability of screening measures for depression and anxiety disorders in multiple sclerosis. Mult Scler Relat Disord 20 (2018) 9-15.

[18] [Eysenbach G Improving the quality of Web surveys: the Checklist for Reporting Results of Internet E-Surveys (CHERRIES). J Med Internet Res (2004) 6:e34](http://paperpile.com/b/X7EYBU/i7Fm).
